# Current State of Breast Cancer Diagnosis, Treatment, and Theranostics

**DOI:** 10.3390/pharmaceutics13050723

**Published:** 2021-05-14

**Authors:** Arya Bhushan, Andrea Gonsalves, Jyothi U. Menon

**Affiliations:** 1 Ladue Horton Watkins High School, St. Louis, MO 63124, USA; abhushan2004@gmail.com; 2 Department of Biomedical and Pharmaceutical Sciences, College of Pharmacy, University of Rhode Island, Kingston, RI 02881, USA; agonsalves@uri.edu

**Keywords:** breast cancer, imaging modalities, mammography, breast specific gamma imaging, triple-negative breast cancer, theranostics

## Abstract

Breast cancer is one of the leading causes of cancer-related morbidity and mortality in women worldwide. Early diagnosis and effective treatment of all types of cancers are crucial for a positive prognosis. Patients with small tumor sizes at the time of their diagnosis have a significantly higher survival rate and a significantly reduced probability of the cancer being fatal. Therefore, many novel technologies are being developed for early detection of primary tumors, as well as distant metastases and recurrent disease, for effective breast cancer management. Theranostics has emerged as a new paradigm for the simultaneous diagnosis, imaging, and treatment of cancers. It has the potential to provide timely and improved patient care via personalized therapy. In nanotheranostics, cell-specific targeting moieties, imaging agents, and therapeutic agents can be embedded within a single formulation for effective treatment. In this review, we will highlight the different diagnosis techniques and treatment strategies for breast cancer management and explore recent advances in breast cancer theranostics. Our main focus will be to summarize recent trends and technologies in breast cancer diagnosis and treatment as reported in recent research papers and patents and discuss future perspectives for effective breast cancer therapy.

## 1. Introduction

Breast cancer has a very long history as it was first reported by the ancient Egyptians more than 3500 years ago in about 1500 B.C [1]. Today, breast cancer is the second most prevalent type of cancer and is a leading cause of most cancer-related deaths in women in the United States [2]. Around 281,550 women are projected to be diagnosed with breast cancer in 2021, and 43,600 women are predicted to die due to breast cancer in the US, according to the American Cancer Society. Early diagnosis of the disease is crucial for effective treatment and positive prognosis, as significantly lower probability of dying and higher survival rate is observed in patients with smaller tumors at the time of diagnosis [3]. Early detection of breast cancer and accurate lesion assessment are, therefore, the primary focus of all imaging modalities. At present the two major pillars to be addressed for effective management of breast cancer disease include: (i) diagnosis of breast cancer in its earliest stages and (ii) providing timely treatment after diagnosis to save lives.

**Figure 1:**
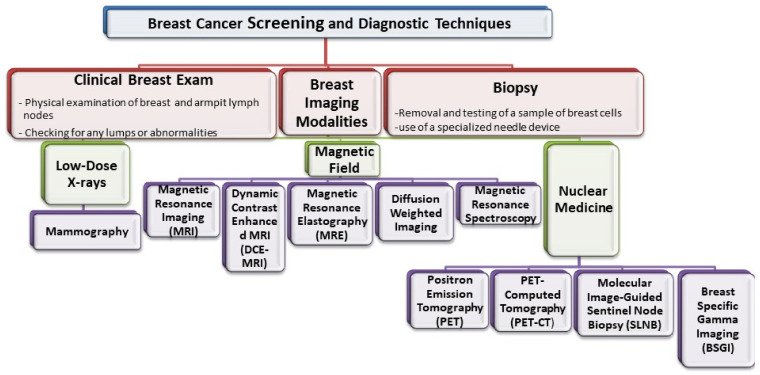
Representation of the various imaging techniques that can be used in breast cancer diagnosis.

Imaging of the breast is utilized almost exclusively for detection, diagnosis, and clinical management of cancers and for the assessment of the integrity of breast implants (Figure 1) [4]. As a conventional medical imaging modality, ultrasound has played a key role in breast cancer detection, image-guided biopsy, and lymph-node diagnosis for many years. Mammography, ultrasonography, magnetic resonance imaging (MRI), scintimammography, single photon emission computed tomography (SPECT), and positron emission tomography (PET) are other commonly used imaging modalities [5,6,7]. Based on the diagnosis and assessment of the extent of breast cancer, the need for preoperative (neoadjuvant) systemic therapy is determined. Targeted and effective therapies with minimal off-target side effects are needed for breast cancer treatment. As breast cancer is a global problem, major emphasis also needs to be put on diminishing worldwide disparities in terms of access to diagnosis, multimodal treatment, and novel drugs.

For this review, we conducted a literature search within the Google Scholar and PubMed databases using the keywords: “Breast Cancer”, “Imaging”, and “Treatment” in the title field, with dates from 2000 to 2021. After reading the abstracts, we manually selected the relevant papers for this review. In this review article, we examine various detection techniques for breast cancer, provide an in-depth analysis on the therapies for different subtypes of breast cancer, and investigate recent trends and the future of breast cancer theranostics.

## 2. Techniques for Diagnosis or Detection of Breast Cancer

Early diagnosis is a key to successful breast cancer treatment. T1 tumors measuring less than 2 cm in diameter have a 10-year survival of approximately 85%, while T3 tumors—essentially the result of delayed diagnosis—have a 10-year survival of less than 60% [8]. Imaging techniques commonly used for detection of breast cancer are summarized in Table 1.

### 2.1. Mammography

A mammogram is an x-ray of the breast that can reveal benign or malignant abnormalities. It is obtained by applying a small dose of radiation through the breast post compression between two plates to produce an x-ray image. Mammograms can be utilized for both screening and diagnosis [31]. Mammogram screening is performed as an attempt to detect any early signs of breast cancer, even before symptoms occur, to decrease mortality by early diagnosis. Diagnostic mammogram assists in detecting breast cancer if a woman experiences symptoms, for instance, a lump that can be felt in her breast [32]. In 2009, new mammography screening guidelines were issued by the U.S. Preventive Services Task Force (USPSTF) with a recommendation that routine screening mammography for women under age 50 is not needed, whereas its earlier stance was in accordance with American Cancer Society guidelines, which recommended mammography every one to two years for all women age 40 and older [31,32,33,34]. In addition, since radiologists assess information subjectively, breast density cannot be utilized to infer the information ingrained in a mammogram [35]. For instance, patients may have appreciably different mammograms, each with vastly different outcomes, but have the same breast density assessment value. In previous studies, mammography results have been used to develop statistics related to glandular tissue volume. However, these automated methods of evaluating breast density are not sufficient to predict breast cancer prevalence [36]. Recently, gold-based nanoformulations have shown promise in significantly enhancing the contrast in mammographic images [37]. Mammographic density can improve the accuracy of breast cancer risk models. More accurate risk prediction can also be achieved by a mammography-based deep learning (DL) model [36].

### 2.2. Magnetic Resonance Imaging

Breast MRI is a non-invasive and non-ionizing diagnostic imaging tool that employs low-energy radio frequency waves and a magnetic field to obtain detailed images of structures within the breast [38]. MRI can be used to measure the size of the cancer and look for metastasized tumors in women who have been previously diagnosed with breast cancer. Tumors with size less than or equal to 2 cm have been accurately identified and measured using MRI. However, larger breast tumors are often overestimated due to the abnormal breast tissue encompassing the actual lesion, which can lead to greater mastectomy rates [39,40]. Goldsmith et al. first described the use of nuclear MRI for the breast 40 years ago [41]. Several uses of MRI for the breast, including screening the high-risk population, have been recommended by the American College of Radiology [42]. MRI has the ability to detect suspected breast malignancies that often escape clinical, mammographic, and ultrasound detection [37]. Fe_3_O_4_, gadolinium(III)-, and Mn(II)-based contrast agents are commonly used for preoperative assessment, especially to visualize axillary lymph nodes of the breast [43]. To reduce the possibility of off-target toxic effects and increase specificity towards breast cancer, these contrast agents may be encapsulated within breast cancer-targeting polymeric carriers [44,45]. Because of the high sensitivity and lower specificity of breast MRI, it is widely used in breast cancer diagnostics, thus, resulting in an increase in incidental findings. It is imperative that these findings be histologically assessed before any surgical intervention [46,47].

### 2.3. Dynamic Contrast Enhanced MRI (DCE-MRI)

Dynamic contrast-agent-enhanced breast MRI works by analyzing the temporal enhancement pattern of a tissue following the intravenous injection of a paramagnetic contrast agent. This non-invasive imaging technique quantitatively determines the extent of tissue vascularization, interstitial space composition, and differentiation of lesions [48]. This imaging modality is useful to depict tumor angiogenesis with overall recurrence and overall survival of breast cancer patients [49,50,51]. As a result, lymph node metastasis that occurs due to greater angiogenesis in breast cancer can also be predicted using this imaging modality. DCE-MRI, when combined with a computer-aided diagnosis technology, such as texture analysis, can also be used to identify estrogen receptor positive (ER+) breast cancer subtypes [52]. DCE-MRI technique is non-invasive and three-dimensional, which allows visualization of the extent of disease before morphological alterations and helps to predict the overall response either before the start of therapy or early during treatment [53,54]. Unlike mammography, DCE-MRI is not limited by breast tissue density. However, a central limitation of DCE-MRI is that it is non-specific [55].

### 2.4. Magnetic Resonance Elastography 

Magnetic resonance elastography (MRE) can be used to obtain details on tissue mechanical properties in vivo [56]. Following application of an external stress, breast MRE, a non-invasive, non-ionizing, and cross-sectional imaging modality, can quantitate the viscoelastic properties of breast tissues [57]. Breast cancers often have a higher stiffness due to increase in the number of cells, collagen, and proteoglycans compared to the normal surrounding tissues and benign lesions [58,59]. Although manual palpation is commonly used for routine screening of the breast, it lacks specificity and sensitivity. This is where the limitations of manual palpation can be overcome by MRE scanning of the breast [60,61,62]. While the initial results are encouraging, the most significant limitation for MRE in breast cancer is spatial resolution and detection of small focal lesions due to the overlap in the soft malignant tumors and stiff benign lesions elasticity ranges [63].

### 2.5. Diffusion-Weighted Imaging

Diffusion-weighted imaging (DWI) is a form of unenhanced MRI that uses the diffusion of water molecules to generate contrast in MR images to address some of the shortcomings faced by regular breast MRI [64,65,66]. The potential benefits of DWI techniques include improved differentiation of benign and malignant breast lesions and assessment and prediction of therapeutic efficacy [67]. DWI has enabled the identification of breast cancer particularly in dense breasts. However, the sensitivity of DWI tends to be variable compared to contrast-enhanced MRI [68]. Technical innovations are helping to overcome many of the image quality issues that have limited widespread use of DWI for breast [69]. While DWI may be an accurate and nonradioactive imaging technique, it has still not achieved its full potential. Detailed investigations and clinical trials are now warranted to prove DWI’s ability to facilitate the diagnostic work-up of the diseases.

### 2.6. Magnetic Resonance Spectroscopy

Magnetic resonance spectroscopy (MRS) can measure a chemical “spectrum” in the region using high magnetic field strengths (typically 11–14 T) on body fluids, cell extracts, and tissue samples, providing additional information about the chemical content in the region [70,71]. The in vivo 1H MRS protocol with the addition of MRI procedure further increases the overall acquisition time by approximately 10 min and has the advantage to improve the diagnostic accuracy of clinical breast MR [72,73]. The MRS specificity has been reported to be approximately 88%; however, the requirement of slightly larger lesions and poor sensitivity to detect total choline (tCho) (a phosphocholine metabolite elevated in breast malignancies and used as a diagnostic biomarker) signal is one of the limitations of this imaging modality [16,74,75]. There has been considerable progress on breast MRS in the last decade; however, multiple factors can potentially limit MRS, like optimization of analysis methods and complexity of acquisition procedures, that need to be addressed before including this imaging modality in a clinical setting.

### 2.7. Positron Emission Tomography (PET) Scanning and PET in Conjunction with Computer-aided Tomography (CT) Scanning (PET-CT)

Positron emission tomography (PET) imaging has been widely adopted as an important clinical modality for oncology. Even though many types of PET radiotracers have been developed to non-invasively interrogate in vivo tumor metabolism, 2-deoxy-2-(18F)fluoro-D-glucose (FDG) is the most widely used US FDA approved PET radiotracer that takes advantage of the enhanced glucose metabolism of cancer cells [76]. Cancerous cells are highly proliferative and have a higher glucose metabolism rate than normal cells. FDG PET radiotracers enter cells via the glucose transporter and are, thus, taken up in greater amounts by tumor cells than by healthy cells [77]. FDG uptake inversely correlates with prognosis [76,77,78].

PET-CT is a combination of PET (a nuclear medicine technique) and CT that produces highly detailed views of the body. The improved spatial resolution and sensitivity of PET scanners dedicated to breast (positron emission mammography) has allowed its clinical application in the study of primary tumors [79,80]. Numerous studies have shown that hybrid imaging with ^18^F-FDG PET/CT provides information about the cellular glucose uptake, which is elevated in malignant lesions [81,82,83,84]. Jørgensen et al. observed significantly reduced uptake of ^18^F-FDG by tumor cells following nanoparticle-assisted photothermal therapy, indicating that it can be effectively used as a marker to assess treatment responses [85]. Physicians use PET-CT studies to diagnose and stage the cancer, plan treatment, evaluate the effectiveness of treatment, and manage ongoing care.

### 2.8. Molecular Image-Guided Sentinel Node Biopsy

Sentinel lymph node biopsy (SLNB) is a revolutionary, minimally-invasive method to determine whether metastasis has occurred in early-stage breast cancer patients. Depending on the nodal metastatic status, SLNB is usually conducted to select the optimal therapeutic approach [86]. SLNB technique is well known for its significantly reduced post-operative complications associated with conventional axillary lymph node dissection [87,88]. This makes effective SLNB management key towards successful breast cancer diagnosis and treatment. Accurate SLNB guidance should limit the amount of invasive procedures needed and determine if multiple-basin drainage is occurring through localizing sentinel lymph nodes, thus, improving the staging accuracy in women with invasive breast cancer [89].

### 2.9. Breast Specific Gamma Imaging

Breast specific gamma imaging (BSGI), a molecular breast imaging approach, is a specialized nuclear medicine imaging test that allows detection of sub-centimeter and mammographically occult breast cancer with a sensitivity and specificity comparable to MRI [26]. In BSGI, a radiotracer such as Technetium Tc99m Sestamibi is injected into the patient’s bloodstream and the breast is visualized using a special camera [90,91,92]. Unlike mammography, BSGI is unaffected by breast density [93,94]. The modern BSGI has improved sensitivity for the detection of sub-centimeter lesions compared to scintimammography [95]. The major drawback of this technique is that, since the whole body gets exposed to the radiation, it is not possible to employ this for frequent breast cancer screening [96].

### 2.10. Ultrasound

Although mammography is a gold standard for breast cancer imaging, because of its limitations regarding dense breasts, another supplementing screening tool is required. Ultrasound is a supplemental tool that may be utilized to analyze some breast changes in women with dense breast tissues, as well as suspicious areas not seen on a mammogram [97]. Advantages of this technique include its wide availability, as well as no patient exposure to radiation. At the same time, however, it is limited by a number of factors. Most notably, it may fail to detect microcalcifications, and it may miss some early signs of cancer. Because of this limitation, this technique is not used to screen for breast cancer and is reserved for special situations. The fusion of ultrasound with other modalities [98,99]. such as ultrasound imaging techniques and ultrasound-guided biopsy, provides important tools for the management of breast cancer patients. Ultrasound elastography is now a routine noninvasive tool used to measure the consistency or hardness of the tissues to differentiate benign and malignant breast lesions [100,101]. Contrast-enhanced ultrasound and other modalities fused with ultrasound are other tools that may be useful in the noninvasive prediction of prognostic factors of breast cancer [102]. Complementary high resolution ultrasound is excellent for detecting breast lesions when in expert hands [103]. On the basis of existing literature, it was found that fusion of other modalities with ultrasound may be an effective primary detection tool for breast lesions, particularly in low- and middle-income countries with low-resource settings and where mammography and other expensive techniques are not available [104].

From the literature discussed above, we can see that, although ultrasound and mammography remain the most commonly used conventional methods of diagnosing breast cancer, other modalities such as DCE-MRI, MRE, PET, PET-CT, SLNB, and BSGI are now being considered for efficient collection of data. For example, most mammography methods can only be used to gather information about one breast, while MRI can be used to collect data from both breasts at the same time. Use of contrast agents can also enhance the quality of the data obtained for breast cancer diagnostics.

## 3. Current Treatment and Novel Therapies for Different Subtypes of Breast Cancer 

**Figure 2:**
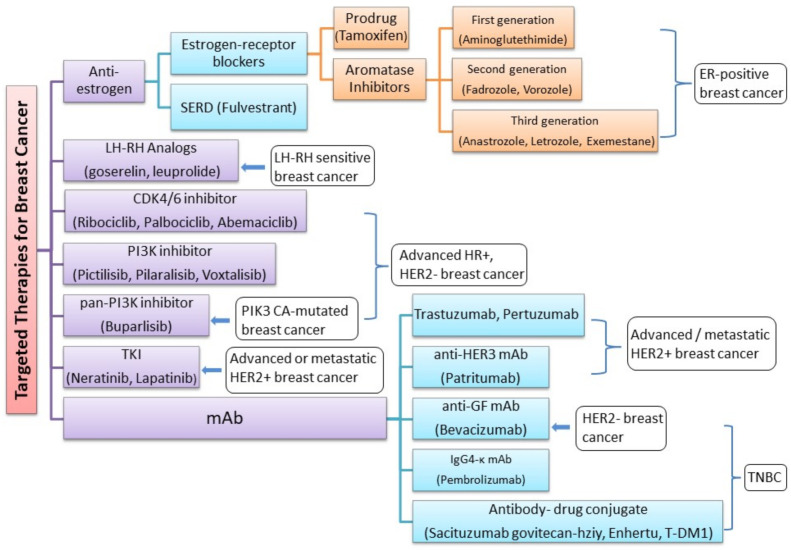
Novel FDA-approved targeted therapies for the treatment of molecular subtypes of breast cancer.

Breast cancer diagnosis by breast examination, mammography, breast ultrasound, MRI, and other imaging modalities can help identify tumors and other abnormalities in the tissue, as described above. These imaging modalities can help find a lump, an area of microcalcification, a suspicious area on ultrasound, or a gadolinium-enhanced area on MRI. Once breast cancer is identified using one of the diagnostic modalities discussed above, immediate and rigorous treatment must be provided to remove the tumor and prevent further spread of the cancer. One of the major challenges for breast cancer treatment is its heterogeneous nature, which affects the response to therapy [105]. By evaluating the presence of biomarkers such as hormone receptors (HRs), excess levels of human epidermal growth factor receptor 2 (HER2) protein, and/or extra copies of the HER2 gene [106,107], treatments that are most effective against a particular type of breast cancer can be determined and administered (Figure 2). Based on the upregulation of genes, there are five main intrinsic or molecular subtypes of breast cancer:

- **Luminal A** breast cancer is low grade, HER2– and HR+ (estrogen- and/or progesterone-receptor positive), that has low levels of the protein Ki-67, which are responsible for controlling how fast cancer cells proliferate. Luminal A cancers tend to proliferate slowly with an excellent prognosis compared to other cancers.
- **Luminal B** breast cancer is a molecular subtype of breast cancer in which the tumors are HR+ (progesterone-receptor and/or estrogen-receptor positive) and show elevated levels of the protein Ki-67 while being either HER2– or HER2+. Luminal B cancer subtype is associated with faster proliferation rate and tends to be more aggressive compared to Luminal A breast cancer, making its prognosis slightly worse [108].
- **Triple-negative/basal-like** breast cancer is HR– (estrogen-receptor and progesterone-receptor negative) and HER2–. Women with *BRCA1* gene mutations are more prone to develop this form of cancer [109].
- **HER2-enriched** breast cancer is a molecular subtype of breast cancer in which tumors are HER2+ and HR– (i.e., negative for estrogen- and progesterone-receptor). This subtype is associated with a tendency to proliferate at a more rapid rate than luminal cancers [91]. However, patients are successfully treated with drugs targeting the HER2 protein, such as Tykerb (lapatinib), Herceptin (trastuzumab), Perjeta (pertuzumab), and Enhertu (fam-trastuzumab-deruxtecan-nxki) [110].
- **Normal-like** breast cancer is identical to luminal A cancer as it is HER2–, HR+ (estrogen- and/or progesterone-receptor positive) with reduced levels of the Ki-67 protein. The prognosis of normal-like breast cancer is, however, slightly worse than the luminal A cancer.

There are several FDA-approved drugs currently used in the treatment of breast cancer (Table 2). The prodrug tamoxifen (brand name: Nolvadex) is a partial agonist that blocks estrogen uptake by the estrogen receptor (ER) [111,112]. Studies have shown that the risk of ER+ breast cancer recurrence can be reduced by half with tamoxifen [113]. However, similar to most anti-cancer therapies, tamoxifen has known side-effects and has been found to be associated with a number of increased health risks, such as endometrial cancer, blood clots, and stroke [114,115]. Aromatase inhibitors (AIs) block estrogen from being produced in postmenopausal women, suppressing the conversion of androgens to estrogens, thus, resulting in estrogen depletion. Three generations of AIs have been developed. The first-generation (e.g.: aminoglutethimide) and second-generation AIs (e.g., fadrozole and vorozole) are less selective with decreased production of cortisol and aldosterone, in addition to aromatase. They are also poorly tolerated with limited clinical efficacy [116]. On the other hand, third-generation AIs (e.g.: anastrozole (brand name: Arimidex), letrozole (brand name: Femara), and exemestane (brand name: Aromasin)) are highly selective for the enzyme aromatase and are tolerated fairly well. As a result, they have surpassed tamoxifen as first-line therapy for postmenopausal women with HR+ metastatic breast cancer with excellent response rates and delayed progression. AIs have additionally shown incremental improvement in disease-free survival, lower local and metastatic recurrence rates, and a lower incidence of contralateral breast cancer over tamoxifen [117].

Luteinizing hormone-releasing hormone (LH-RH) analogs (goserelin and leuprolide) suppress the production of hormone from the ovaries [121]. LH-RH agonists act by pituitary desensitization and receptor downregulation, thereby suppressing gonadotrophin release. LH-RH exerts direct anticancer activity on malignant tissue that is independent from the suppression of the ovarian steroid synthesis and secretion [145,146]. Fulvestrant, a selective estrogen receptor degrader (SERD), is another drug that is suitable for breast cancer patients refractory to previous hormonal therapy. This is the first selective ER down regulator that is available clinically. This pure anti-estrogen results in degradation of ER alpha (α), has no agonistic effects, and has also demonstrated activity in tamoxifen-resistant breast cancer models [122]. Fulvestrant is the first SERD to enter into the clinical arena and a suitable backbone for combination therapy with new targeted agents for endocrine treatment of breast cancer. Preclinical studies have demonstrated that fulvestrant downregulates the expression of ERα in ER+ breast cancer cell lines without decreasing ERα gene (ESR1) transcripts along with inhibition of ER-responsive genes [147]. Fulvestrant can additionally block the non-genomic actions of estradiol on the G-protein coupled estrogen receptor (GPER), an alternate ER with a structure distinct from the two canonical ERs (ERα and ERβ) that is expressed in 50–60% of breast cancer, and which has been surmised to be related to the development of resistance towards tamoxifen in ERα+ breast cancer patients [148]. The proliferation of ER+ breast cancer cells is prevented through these processes. Additionally fulvestrant is also effective in those cell lines that are resistant to tamoxifen [149,150]. Patient derived xenograft models of ER+ breast cancer corroborated fulvestrant’s antitumor activity. Thus, we can conclude that it is more efficacious compared to tamoxifen or estrogen withdrawal [151]. Endocrine drugs work by different mechanisms, and thus, they are usually used as a combinational therapy for better anticancer efficacy. Nevertheless, there are conflicting results reported. It is generally believed that patients with endocrine-therapy-naïve advanced breast cancer and those with highly endocrine-sensitive tumors may benefit the most from combination endocrine therapy [152,153,154,155]. Several other biomarkers have emerged as potential targets for breast cancer therapy as described below.

### 3.1. Cyclin-Dependent Kinases 4/6 (CDK4/6) Pathway

CDK4/6 are pivotal drivers for cell proliferation as they combine with cyclin D proteins, which regulate cell processes during the G1 phase of the cell cycle. Complete understanding of this cell cycle regulation may lead to promising cancer therapies [124]. Numerous studies are being carried out to explore drugs inhibiting CDK4/6 and assess the efficacy and drug safety for the treatment of breast cancer [156]. As a result of severe adverse events and less activity, the development of pan-CDK inhibitor flavopiridol [157] was subsequently discontinued, and then, highly specific inhibitors, namely, ribociclib (LEE011), palbociclib (PD0332991), and abemaciclib (LY2835219), were extensively researched and developed [124,125,126]. US FDA has approved palbociclib and ribociclib for the treatment of HR+, HER2–, or metastatic breast cancer. Recent clinical trial data suggest that significantly improved clinical outcome of palbociclib was achieved when combined with letrozole or fulvestrant [158,159,160,161].

### 3.2. Phosphoinositide 3-kinase (PI3K) Pathway

The PI3K pathway, also called phosphatidylinositol 3-kinases, is the most commonly activated signaling pathway in human cancer. They are a family of enzymes that are involved in cellular functions linking oncogenes and multiple receptor classes and constitute a critical signal transduction system [162]. The phosphatidylinositol-3-kinase (PI3K)/AKT/mammalian target of rapamycin (mTOR) pathway (PI3K/AKT/mTOR pathway) plays a key role in cancer [126]. Pan-PI3Ki bind to PI3K isoforms in a selective and ATP competitive manner. The combination of PI3K inhibitors with aromatase inhibitors has been used as second-line treatment for HR+/HER− advanced breast cancer. A potent and highly specific oral pan-class I PI3K inhibitor (pan-PI3Ki), buparlisib is currently under investigation in patients with a variety of solid tumors, including breast cancer [127,128]. According to a new study, toxicities associated with buparlisib make it a poor option for the treatment of patients with HR+, HER2– advanced breast cancer that progressed on or after mTOR inhibitor therapy. The efficacy of the agent, however, suggests that PI3K inhibitors, along with endocrine therapy, remain a reasonable approach in patients with PIK3CA mutations [128].

Another pan-PI3Ki that displays equipotent inhibition of the p110α and –δ PI3K isoforms and less potent inhibition of p110β and –γ isoforms is pictilisib [129]. In a phase I dose-escalation clinical trial of 60 patients with advanced solid tumors (NCT00876109), pictilisib was found to be overall safe in patients but with severe side-effects, such as hyperglycemia, rash, and pneumonitis [140]. Additionally, pilaralisib, also known as XL147, is an orally bioavailable small molecule with potential antineoplastic activity [131]. XL147 selectively targets and binds reversibly to class 1 PI3Ks thereby inhibiting tumor cell proliferation within tumors that are susceptible. Tumorigenesis is often related to the activation of the PI3K signaling pathway. In a Phase I/II dose-escalation study, pilaralisib (SAR245408), or voxtalisib (SAR245409), a PI3K and mammalian target of rapamycin inhibitor, in combination with letrozole, was evaluated for its efficacy, safety, and pharmacokinetics in HR+, HER2–, non-steroidal AI-refractory, recurrent, or metastatic breast cancer. As compared to voxtalisib, patients who were administered with pilaralisib demonstrated increased glucose levels compared to those who were administered voxtalisib. In conclusion, a limited efficacy and an acceptable safety profile in endocrine–therapy-resistant HR+, HER2– metastatic breast cancer was observed in patients treated with pilaralisib or voxtalisib combined with letrozole, as shown [132].

### 3.3. Targeting HER2+ Breast Cancers

HER2+ breast cancer (HER2+ BC) is characterized by drug resistance and a high rate of metastasis. Targeted therapy drugs have been shown to greatly improve the prognosis of HER2+ BC patients, but drug resistance or severe side effects have limited the clinical application of targeted therapy drugs. Various strategies are being researched to overcome drug resistance and to attain a more effective treatment. The HER2 oncogene (HER2, HER2/neu, c-erbB-2) is situated on chromosome-17 [163,164], and the main function of this oncogene is to encode transmembrane receptor tyrosine kinase [165]. Tyrosine kinase receptors play a key role in mediating various cellular functions, such as cell motility, proliferation, metabolism, and differentiation, that are based on cell-to-cell communication [140]. These receptors consist of a singular transmembrane helix, extracellular ligand domain and an intracellular region of a tyrosine kinase domain, juxtamembrane region, and a carboxy terminal tail [140]. Tyrosine kinase inhibitors competitively inhibit tyrosine phosphorylation and block tyrosine kinase enzyme activity, thus, resulting in downregulation of many cellular functions [166]. Neratinib (NERLYNX, Puma Biotechnology, Inc., CA, USA), an irreversible tyrosine kinase inhibitor (TKI) of HER1/HER2/HER4, has been reported to significantly improve the 2-year invasive disease-free survival after trastuzumab-based adjuvant therapy in HER2+ BC [137]. Neratinib, in combination with capecitabine, was approved by the US FDA on 25 February 2020 for treating patients with advanced or metastatic HER2+ BC previously treated with two or more anti-HER2 based regimens in the metastatic setting. Another example of TKI is Lapatinib, which competitively inhibits ATP-binding sites intracellularly and reversibly blocks phosphorylation of HER1 and HER2 [167]. A phase III study of lapatinib, in combination with an anti-neoplastic drug paclitaxel, demonstrated an increase in the survival rate of patients with HER2 metastasis breast cancer [168]. Another drug moiety, tucatinib, exhibited greater selectivity for HER2 in a phase I study of advanced disease patients along with reduced occurrence of diarrhea, as reported by patients that received other TKIs [169].

Compared with HER2– tumors, HER2+ BC is an aggressive subtype that demonstrates unique epidemiological, clinical, and prognostic differences with poor response to standard chemotherapy regimens [170]. About 30% of breast cancer patients have been evaluated for the expression of HER2, which is generally recognized as a marker for invasive disease that is likely to be highly metastatic, drug resistant, and to spread rapidly [171,172,173]. There has been remarkable advancements in therapies for managing HER2+ BC in the last 20 years, specifically, targeted treatments that are HER2 expression level dependent [174]. A humanized monoclonal antibody (mAb), trastuzumab (herceptin), targeted towards the HER2 ectodomain, has demonstrated activity in HER2-overexpressing breast cancer patients. Trastuzumab effectively inhibited basal and induced HER2 cleavage, resulting in the generation of phosphorylated p95 [171]. Another mAb, pertuzumab, binds to a different epitope of the HER2 dimerization domain than trastuzumab, preventing interactions with other receptors in the HER2 family that lead to cell growth inhibition [175,176]. The direct inhibitory action on the extracellular domain of HER2 has largely contributed to the HER2-directed mAbs antitumor efficacy.

Patritumab, a human anti-HER3 mAb, through inhibiting the formation of HER2/HER3 heterodimers, has shown anticancer activity in preclinical models. It was found to exhibit favorable efficacy and acceptable tolerability in patients with HER2+ advanced breast cancer [138]. The pharmacokinetic profile for patritumab was determined based on the target trough level, and efficacy was evaluated based on the overall response rate and progression-free survival.

### 3.4. Treating Triple-Negative Breast Cancer

Triple-negative breast cancer (TNBC) accounts for about 10–15% of all breast cancers [177]. In TNBC, the cancer cells do not possess estrogen or progesterone receptors and also do not produce too much of the protein HER2 [178]. As compared to other breast cancer subtypes, TNBC is far more invasive and proliferate and spreads at a much faster rate, and patients have limited treatment options and a worse prognosis [179,180]. Standard chemotherapy remains the mainstay treatment for TNBC. However, metastasis and recurrence rates are higher compared to non-TNBC tumors [181]. Advanced TNBC patients, when treated with carboplatin with or without a taxane drug (e.g., docetaxel), showed better efficacy and toxicity profile compared to docetaxel. Additionally, in germline BRCA1/2-mutated breast cancer patients, carboplatin displayed a response rate twice as high compared to docetaxel. This implies the importance of determining whether breast cancer patients have BRCA1/2 mutation so that the most effective drug for first-line chemotherapy can be chosen [182]. TNBC has the fewest therapeutic options among all breast cancer subtypes due to the lack of well-defined molecular targets [181]. Identification of new therapeutic targets and development of effective targeted agents is, hence, urgently needed.

Sacituzumab govitecan is the first antibody–drug conjugate approved by the US FDA in the treatment of relapsed or refractory metastatic TNBC. It was developed by coupling a monoclonal antibody that targets anti-trophoblast cell-surface antigen 2 (Trop-2) with SN-38—an active metabolite of irinotecan, which is a topoisomerase I inhibitor. Approval was based on findings in the phase I/II multicenter IMMU-132-01 trial (ClinicalTrials.gov identifier NCT01631552) [183]. Another drug, enhertu, is an antibody and topoisomerase inhibitor conjugate that targets and attaches to HER2+ cancer cells [142]. Enhertu is approved for treating adults with unresectable or metastatic HER2-positive breast cancer [184].

Kadcyla, also known as T-DM1, is an agent approved by the US FDA to treat patients with HER2-positive metastatic breast cancer that have been previously treated with herceptin and taxane chemotherapy (neoadjuvant treatment). T-DM1 is an antibody–drug conjugate targeted therapy in which emtansine is conjugated to Herceptin [141].

The immunotherapy medicine pembrolizumab (brand name: Keytruda) is a human monoclonal IgG4-ĸ antibody that is highly selective against the programmed cell death 1 receptor (PD-1). The addition of pembrolizumab to first-line chemotherapy significantly extended progression-free survival among patients with metastatic TNBC or TNBC that has resurged and cannot be surgically removed [143]. Recently, USFDA granted accelerated approval of pembrolizumab in combination with chemotherapy for treating TNBC patients.

Recently, the combination of atezolizumab plus nab-paclitaxel has been approved by FDA as first-line therapy in patients with PD-L1+ TNBC [144]. We can, therefore, see that several diagnostic/ imaging and therapeutic options are currently available for breast cancer management. There has been increasing interest in recent times to combine diagnostic and therapeutic components within a single system for effective and personalized breast cancer management. Strategies being investigated in this direction are described in the next section.

## 4. Recent Trends in Breast Cancer Theranostics

Traditionally, cancer management is based on identifying tumor lesions through an appropriate diagnostic imaging modality, followed by treatment with chemotherapy, radiotherapy, or surgery. However, the disadvantages of these treatments include possibility of incomplete surgical resection, off-target toxicities, low local drug concentrations at the disease site, and limited drug penetration into tumors due to abnormal vasculature, which causes elevated interstitial pressure and blood flow stasis [185,186]. Moreover, conventional methods of assessing drug kinetics involves assessing drug concentration in plasma, which is not a reliable method to evaluate chemotherapeutic pharmacokinetics [187,188]. Over the past two decades, personalized medicine has received significant interest as it can be used to tailor treatment according to patient needs and characteristics, thus, minimizing side-effects, resulting in the emergence of theranostics, which is a relatively new research area [189]. Theranostics is a field of research where a combination of diagnostic agents and therapeutic agents are used to provide patient-centered care for the treatment of cancer and other diseases by providing real-time monitoring of the drug that will assist in altering cancer treatment regimens for better therapeutic efficacy [190]. Accurate diagnosis is crucial for an early therapeutic intervention, failure of which results in delayed treatment and increased risk of mortality [191].

**Figure 3:**
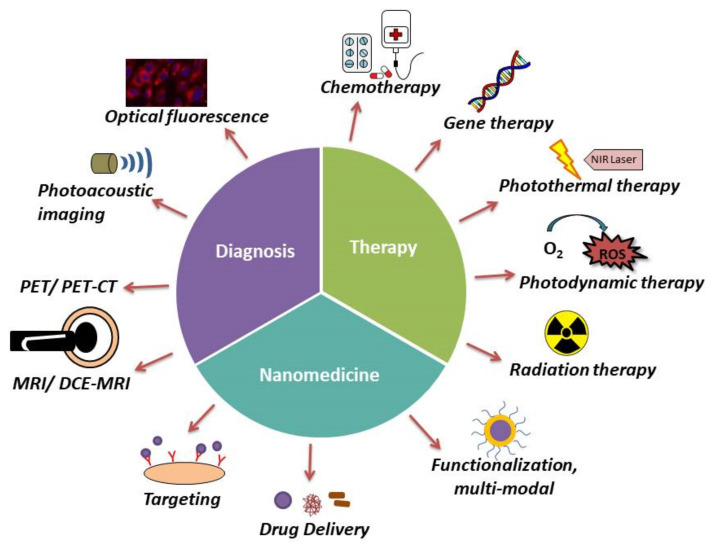
Schematic diagram representing theranostic approaches in breast cancer management.

Theranostic nanotechnology or nanotheranostics is an area where an integrated nanotherapeutic system can be used to simultaneously diagnose, deliver targeted therapy, and monitor the therapeutic response [192]. A single nanoparticle formulation, conjugated with targeting ligands, therapeutic agents, and a fluorophore/contrast agent, can be visualized using different imaging modalities as it crosses biological barriers to target receptors upregulated by cancer cells and finally releases the drug in the tumor environment in a controlled manner (Figure 3).

Nanotheranostics is being widely explored today as a method of effectively managing breast cancer. Nanotheranostic formulations can be tracked using different imaging modalities following administration so that their targeted accumulation and treatment at the site of the cancer can be monitored. Lipid-based carriers, such as liposomes and micelles, are often used due to their versatility and biocompatibility. Gregoriou et al. recently developed theranostic micelles using Pluronic F127 block copolymer and Vitamin E-TPGS that showed promise as a method of targeted delivery of the phytochemical resveratrol to treat breast cancer. Coumarin-6—a fluorophore, can be incorporated to impart imaging capabilities to the system [193]. Wang et al. targeted EGFR+ TNBCs using a quantum-dot-containing micellar formulation tagged with an anti-EGFR nanobody. The micelles could be imaged using the near-infrared fluorescent quantum dots and could release the anti-cancer drug aminoflavone. Significant tumor regression was observed in orthotopic TNBC mouse models with EGFR+ tumors following administration of the theranostic micelles by i.v. injection [194]. Parhi et al. functionalized lipid-based NPs with trastuzumab to target HER2+ breast cancer cells. The NPs (~72 nm) contained rapamycin (anti-cancer drug) and quantum dots (imaging). In vitro studies on SKBR 3 breast cancer cells grown as a two-dimensional monolayer and as three-dimensional spheroids confirmed greater cellular uptake and therapeutic efficacy than native drug or unmodified NPs [195]. Albumin NPs have also been investigated as a delivery vehicle for theranostic applications. A human serum albumin-based NP formulation (~151 nm) encapsulating doxorubicin (DOX, chemotherapeutic drug) and gadolinium III (MRI contrast agent) was developed recently and studied against TNBC xenografts grown on the chorioallantoic membrane of fertilized chick eggs. Persistent NP presence was observed in tumor tissues for at least 15 h, where the NPs significantly reduced the proliferative Ki-67-positive fraction of cells in the xenografts compared to native DOX [196].

Theranostic formulations developed using different polymers have also been successfully investigated in the treatment of breast cancer and its metastases. For example, Li et al. developed a novel terpolymer using poly(methacrylic acid) and PS 80 covalently grafted onto starch, which was then used to deliver DOX and multiple imaging agents—gadolinium (MRI contrast agent) and near-infrared fluorophore HF750 (fluorescence imaging), for the treatment of brain metastases of breast cancer. The NPs, when delivered by tail vein injection, could selectively accumulate and induce apoptosis in cancer cells while not affecting normal brain cells in a brain metastatic breast cancer SCID mouse model [197]. Poly lactic-co-glycolic acid (PLGA) is a polymer that has been FDA-approved for many medical applications and is widely used in nanoparticle-based drug delivery strategies. Recently, PLGA NPs were developed and coated with platelet membranes to form nanoplatelets containing DOX, as well as multiple imaging agents—perfluoropentane (PFP for ultrasonic imaging), nanocarbon (for photoacoustic imaging and photothermal therapy), and fluorescence imaging. Upon delivery of the NPs to 4T1 breast-tumor-bearing mice and laser irradiation, the light was converted to heat energy by the NPs, which had a photothermal effect. The heat also led to PFP vaporization for enhanced ultrasonic imaging and release of DOX for therapy [198]. Dong et al. were able to successfully develop a dual-modal gold-nanoshelled PLGA magnetic hybrid nanoparticle formulation that was encapsulated with perfluorooctyl bromide and superparamagnetic iron oxide nanoparticles and conjugated to anti-HER2 antibodies (HER2-GPH NPs). They were able to monitor the accumulation of these particles using ultrasound and magnetic resonance while the targeted antibody aided the binding of photothermal agents to the HER2-positive breast cancer cells. These particles were able to successfully induce cell death on exposure to near-infrared irradiation [199].

Metal-based NPs have also been explored in breast cancer nanotheranostics. Ruthenium (Ru) agents also display high anti-cancer activity with limited cytotoxicity towards normal cells and are, therefore, an attractive alternative to platinum-based compounds for anti-cancer therapy [200]. Ru-based compounds can also be employed as imaging agents by binding to the DNA through non-covalent interactions [201] and are, thus, useful tools in theranostic applications. Shen et al. reported the development of a liposome-based theranostic formulation containing Ru-polypyridine complex. The liposome carrier enhanced the cellular internalization of Ru in cancer cells. Intravenous (i.v.) administration of these nanocarriers in orthotopic murine model of MDA-MB-231 human breast cancer exhibited high accumulation of the particles within the tumor 2 h post injection, along with a dramatic decrease in the TNBC tumor growth [202]. We have previously developed theranostic nanoformulations that can co-deliver a ruthenium compound (therapy) along with a radionuclide (imaging) to epidermal growth factor (EGFR)-positive cancer cells [103]. This formulation is also suitable for the treatment of TNBCs, which tend to overexpress EGFR.

In addition to cancer cells, the tumor microenvironment also consists of several other cell types, including fibroblasts and immune cells, that can play a decisive role in the effective distribution of the NPs within the tumor. Strategies that allow for the penetration of NPs into the tumor microenvironment are, therefore, attractive. Zeng et al. developed novel HER2-DOX-superparamagnetic iron oxide nanoparticles (NP) with a gold shell as a theranostic approach for the diagnosis and targeted identification of HER2+ BC. The accumulation of these particles in the tumors of BT474 breast cancer nude mice was highest after 2h of i.v. injection, which was detected by MRI. Additionally, the gold shell-mediated photothermal effect led to remodeling of the tumor microenvironment by decreasing cancer-associated fibroblasts, which resulted in the improved antitumor efficacy of DOX [203]. NPs usually tend to accumulate in tumor tissues as a result of enhanced permeability and retention effect exerted by long-circulating nanoparticles. However, the size of the particles plays a critical role in maintaining these properties. Small nanoparticles easily penetrate deep into tumor lesions; however, they are pulled back into the blood stream during circulation, while large particles, on the other hand, are retained easily but tend to have poor penetration ability [204]. Liu et al. successfully developed a CD44 targeted tumor-specific hyaluronidase-degradable hyaluronic acid, cationic bovine serum albumin-protected gold nanocluster that was loaded with indocyanine green for tumor fluorescence imaging and a chemotherapeutic drug paclitaxel. On subcutaneous injection of the NPs in tumor-bearing Balb/c mice, these particles displayed size-reducible properties as a result of the presence of hyaluronidase leading to highly homogenous intra-tumor distribution of the NPs [205,206].

Nanotheranostic formulations can also be used to provide hyperthermia in cells to promote cell membrane permeabilization causing the destruction of the tumorous mass. Burke et al. used near infrared stimulation of multiwalled carbon nanotubes for photothermal therapy, which led to increased permeability of cell membranes and rapid cell death. This system has the potential to be used for theranostic applications if combined with an anti-cancer agent [207]. Another promising strategy in theranostics is using an injectable thermoresponsive hydrogel for local therapy of breast cancer. Wu et al. demonstrated that injecting a supramolecular thermoresponsive hydrogel such as poly(*N*-acryloyl glycinamide-*co*-acrylamide) hydrogel along with polydopamine (PDA) coated-gold nanoparticles (AuNPs) and loading the carrier with DOX exhibited an excellent photothermal effect, along with sustained release of the anticancer drug [208].

It is clear from the above research that breast cancer nanotheranostics is a rapidly growing area that holds great promise as a method of combining cutting-edge technologies within a single platform to deliver breast cancer therapies in a targeted, sustained, and effective manner. We can integrate contrast agents for different imaging modalities and an anti-cancer therapeutic agent into a single formulation for targeted theranostic drug delivery, which can minimize patient discomfort while providing personalized medicine.

## 5. Conclusions and Future Outlook

In this review, we have highlighted some of the common methods of breast cancer diagnosis and treatment and the role of the emerging area of breast cancer theranostics in integrating diagnostics and therapy within a single platform to provide patient-specific therapy. Early detection and treatment of breast cancer is crucial in the reduction of breast cancer mortality rate. The methods of diagnosis and treatment of breast cancer has undergone tremendous changes over the past two decades, and the focus is on managing and treating the disease with minimal patient discomfort, increased patient compliance, and reduced off-target side effects. Nanomedicine allows for the targeted delivery and controlled release of the encapsulated drugs at the tumor site, thus, altering the bioavailability and drug pharmacokinetics while simultaneously enhancing permeability and retention in the tumor and minimizing severe side-effects to the healthy cells. Theranostics has emerged as an invaluable tool in personalized medicine as these multifunctional platforms can be used for the simultaneous detection, treatment, and management of cancers. Despite the undeniable potential of nanotheranostic formulations, there are several factors to be taken into consideration while developing and testing these platforms and before taking them into the market. A major challenge is in the manufacturing, scale-up, and reproducibility owing to the complexity of incorporating multiple functionalities into a single platform while maintaining its dimensions in the nanoscale range. Extensive research needs to be conducted to determine the optimal dose that can simultaneously produce a strong signal for imaging while maintaining the desired drug release kinetics for therapy. The platform must also have minimal or negligible toxic interactions with the surrounding biological tissues. Depth of penetration is a significant challenge when using imaging modalities with theranostic formulations; therefore, imaging agents that can be used to obtain high resolution images independent of tissue depth are preferred. The materials used to develop the theranostic system must be optimized to prevent release of the incorporated imaging agent and premature release of the encapsulated therapeutics. While stimuli-responsive “smart” materials may be used for on-demand release of therapeutics in response to changes in the surrounding environment (e.g., temperature, pH, magnetic field), this introduces more complexity to the system and can possibly impede its clinical translation. Different type of breast tumors can upregulate different receptors on their surfaces, and the theranostic system will need to be optimized against each type of breast cancer in order to provide personalized therapy. Nevertheless, it is evident that theranostic nanomedicine holds tremendous potential for breast cancer diagnosis and targeted, personalized treatment. Since theranostics is an emerging research area, we can expect to see new multifunctional formulations enter clinical trials in the near future that can be tracked following administration and provide targeted and effective breast cancer therapy.

## Figures and Tables

**Table 1:** Summary of various imaging modalities for screening of breast cancer.

Imaging Modality	Principle	Diagnostic Accuracy	Advantages	Limitations	References
Mammography (First-line tool for breast screening)	Low-dose ionizing x-ray creates detailed images of the breast.	Sensitivity: 75–90% Specificity: 90–95% Spatial Resolution: 50 µm	Most cost-effective. Good response with high specificity and sensitivity. Portable device.	Uses ionizing radiation. Sensitivity decreases with increasing breast density. Accuracy is low in young women. High false-positive results in young women due to dense breasts. Poor contrast compared to MRI.	[9,10,11,12]
Magnetic Resonance Imaging	Uses low-energy radio waves and strong magnets to obtain detailed images of structures within the breast	Sensitivity: 75–100% Specificity: 83–98.4% Spatial Resolution: 25–100 µm	Ability to detect breast malignancies that often escape from clinical, mammograms, and ultrasound detection.	Expensive, inability to standardize the test. Unnecessary breast biopsies due to inability to distinguish between malignant and benign lesions.	[13,14]
Magnetic Resonance Spectroscopy	Employs magnetic field on body fluids and tissue samples to obtain chemical information of that region	Sensitivity: 93% Specificity: 70% Spatial Resolution: up to 0.25 cm^3^	Overcomes limitations of mammography. Radiation-free imaging technology. Excellent Sensitivity. Excellent spatial resolution. All imaging planes possible.	Expensive and time-consuming. Low specificity. Not portable. False-positive results in some benign tumors.	[15,16,17,18]
Dynamic Contrast Enhanced MRI (DCE-MRI)	Multiple MRI scans taken post i.v. injection of contrast agent	Sensitivity: 89–99% Specificity: 37–86% Spatial Resolution: 25–100 µm	Exhibits good performance in monitoring response post therapy.	False-negative results observed due to artifacts based on bleeding and tumor structure.	[14,19,20,21]
Diffusion-Weighted Imaging	Employs diffusion of water molecules to generate contrast.	Sensitivity: 83% Specificity: 84% Spatial Resolution: 25–100 µm	Non-radioactive imaging technique	Failure to detect high water content malignant lesions due to high apparent diffusion coefficients.	[14,22]
MR Elastography (MRE)	Dynamic elasticity imaging technique that combines MRI imaging with low frequency. Employs mechanical waves to create an elastogram to assess tissue stiffness.	Sensitivity: 90–100% Specificity: 37–80% Spatial Resolution: 25–100 µm	Non-invasive, non-ionizing and cross-sectional imaging modality	Lacking in spatial resolution and detection of small focal lesions.	[14,23,24]
Positron Emission Tomography conjugated with computed Tomography (PET-CT)	Combines nuclear medicine technique and computed tomography resulting in high detailed images.	Sensitivity: 90–100% Specificity: 75–90% Spatial Resolution: 2–10 mm	Non-invasive. Provides twice the diagnostic benefits (Elevated activity within the body detected by PET scan and intricate images of tissues and organs by CT scan).	High-cost. Unable to detect tumors less than 8 mm.	[14,23]
Sentinel lymph node biopsy (SLNB)	Surgical procedure to detect spreading of cancer in lymphatic system.	Sensitivity: 90.5% Specificity: 85.7% Spatial Resolution: Not Applicable	Significantly reduces post-operative complications	Not useful for patients with locally advanced cancers and inflammatory breast cancer.	[24,25]
Breast Specific Gamma Imaging	Employs use of a radiotracer. Image captured using a special camera.	Sensitivity: 90–96% Specificity: 71–80% Spatial Resolution: ≥7 mm	Able to identify smaller lesions (<1 cm)	High radiation dose. Not suited for routine tumor screening.	[26,27,28]
Ultrasound	Employs sound waves to image breast tissues	Sensitivity: 80–89% Specificity: 34–88% Spatial Resolution: 50–500 µm	Accessible, real-time lesion visualization, cost-effective, patient compliant.	Failure to detect microcalcifications, possibility of false-positives.	[14,29,30]

**Table 2:** List of therapeutic drugs used in the treatment of different types of breast cancer, and their status.

	Drug	Drug Class	Subtype of Breast Cancer Treated	Status	References
1	Tamoxifen (Brand name: Nolvadex)	Anti-estrogen	ER-positive breast cancer	Approved	[111,112,113]
2	Aminoglutethimide, Fadrozole and Vorozole	First- and second-generation AIs	ER-positive breast cancer	Approved	[116]
3	Anastrozole (Brand name: Arimidex)	Third generation AIs	ER-positive breast cancer	Approved	[118,119,120]
4	Letrozole (Brand name: Femara)				
5	Exemestane (Brand name: Aromasin)				
6	Goserelin and Leuprolide	-	LH-RH sensitive breast cancer	Approved	[121]
7	Fulvestrant	SERD Degrader	Breast cancer	Approved	[122,123]
8	Ribociclib (LEE011)	CDK4/6 inhibitor	Epidermal growth factor receptor 2-negative advanced or metastatic breast cancer	Approved	[124,125,126]
9	Palbociclib (PD0332991)			Approved	
10	Abemaciclib (LY2835219)			Approved	
11	Buparlisib	pan-PI3Ki	(HER2)-negative, PIK3CA-mutated, advanced or metastatic breast cancer	Approved	[127,128]
12	Pictilisib	PI3K inhibitor	HR+/HER− advanced breast cancer	Phase I clinical trial	[129,130]
13	Pilaralisib (XL147)			Phase I/II dose-escalation study	[131,132]
14	Voxtalisib			Phase I/II dose-escalation study	[132]
15	Trastuzumab (Herceptin)	mAb	HER2-overexpressing breast cancer	Approved	[133,134,135]
16	Pertuzumab			Approved	[136]
17	Neratinib	TKI	advanced or metastatic HER2+ breast cancer	Approved	[137]
18	Patritumab	anti-HER3 mAb	HER2+ advanced breast cancer	Preclinical models	[138]
19	Bevacizumab	anti-GF mAb	TNBC patients with germline mutations/ HER2-negative breast cancer	Approved	[139]
20	Sacituzumab govitecan-hziy	Antibody–drug conjugate	Relapsed or refractory metastatic TNBC	Approved	[140]
21	T-DM1 (Kadcyla)	Antibody–drug conjugate	HER-2 metastatic prescription adjuvant treatment when the patient has taken neoadjuvant treatment with trastuzumab (Herceptin) and a taxane	Approved	[141]
22	Enhertu	Antibody–drug conjugate	HER-2 metastatic that has resurged and cannot be removed surgically	Approved	[142]
23	Pembrolizumab (Brand name: Keytruda)	IgG4-ĸ mAb	metastatic TNBC or TNBC that has resurged and cannot be surgically removed	Approved	[143]
24	Atezolizumab combination with nab-paclitaxel	mAb	PD-L1+ TNBC	Approved	[144]

## Data Availability

Not applicable.

## References

[1] Ebeid N.I. (1999). Egyptian Medicine in the Days of the Pharaohs.

[2] Siegel R.L., Miller K.D., Fuchs H.E., Jemal A. (2021). Cancer Statistics, 2021. CA A Cancer J. Clin..

[3] Duncan W., Kerr G.R. (1976). The curability of breast cancer. Br. Med. J..

[4] Juanpere S., Perez E., Huc O., Motos N., Pont J., Pedraza S. (2011). Imaging of breast implants-a pictorial review. Insights Into Imaging.

[5] Basilion J. (2001). Breast imaging technology: Current and future technologies for breast cancer imaging. Breast Cancer Res..

[6] Iranmakani S., Mortezazadeh T., Sajadian F., Ghaziani M.F., Ghafari A., Khezerloo D., Musa A.E. (2020). A review of various modalities in breast imaging: Technical aspects and clinical outcomes. Egypt. J. Radiol. Nucl. Med..

[7] Zhang X.-H., Xiao C. (2018). Diagnostic Value of Nineteen Different Imaging Methods for Patients with Breast Cancer: A Network Meta-Analysis. Cell. Physiol. Biochem..

[8] Sant M., Allemani C., Berrino F., Coleman M.P., Aareleid T., Chaplain G., Coebergh J.W., Colonna M., Crosignani P., Danzon A. (2004). Breast carcinoma survival in Europe and the United States. Cancer.

[9] Zeeshan M., Salam B., Khalid Q.S.B., Alam S., Sayani R. (2018). Diagnostic Accuracy of Digital Mammography in the Detection of Breast Cancer. Cureus.

[10] von Euler-Chelpin M., Lillholm M., Vejborg I., Nielsen M., Lynge E. (2019). Sensitivity of screening mammography by density and texture: A cohort study from a population-based screening program in Denmark. Breast Cancer Res..

[11] Devi R.R., Anandhamala G. (2018). Recent Trends in Medical Imaging Modalities and Challenges for Diagnosing Breast Cancer. Biomed. Pharmacol. J..

[12] Procz S., Roque G., Avila C., Racedo J., Rueda R., Santos I., Fiederle M. (2020). Investigation of CdTe, GaAs, Se and Si as Sensor Materials for Mammography. IEEE Trans. Med. Imaging.

[13] Mann R.M., Kuhl C.K., Moy L. (2019). Contrast-enhanced MRI for breast cancer screening. J. Magn. Reson. Imaging.

[14] Wallyn J., Anton N., Akram S., Vandamme T.F. (2019). Biomedical Imaging: Principles, Technologies, Clinical Aspects, Contrast Agents, Limitations and Future Trends in Nanomedicines. Pharm. Res..

[15] Grover V.P.B., Tognarelli J.M., Crossey M.M.E., Cox I.J., Taylor-Robinson S.D., McPhail M.J.W. (2015). Magnetic Resonance Imaging: Principles and Techniques: Lessons for Clinicians. J. Clin. Exp. Hepatol..

[16] Fardanesh R., Marino M.A., Avendano D., Leithner D., Pinker K., Thakur S.B. (2019). Proton MR spectroscopy in the breast: Technical innovations and clinical applications. J. Magn. Reson. Imaging.

[17] Hu J., Feng W., Hua J., Jiang Q., Xuan Y., Li T., Haacke E.M. (2009). A high spatial resolution in vivo 1H magnetic resonance spectroscopic imaging technique for the human breast at 3 T. Med. Phys..

[18] Alam M.S., Sajjad Z., Hafeez S., Akhter W. (2011). Magnetic resonance spectroscopy in focal brain lesions. J. Pak. Med. Assoc..

[19] Cai H., Liu L., Peng Y., Wu Y., Li L. (2014). Diagnostic assessment by dynamic contrast-enhanced and diffusion-weighted magnetic resonance in differentiation of breast lesions under different imaging protocols. BMC Cancer.

[20] Jansen S.A., Fan X., Karczmar G.S., Abe H., Schmidt R.A., Giger M., Newstead G.M. (2008). DCEMRI of breast lesions: Is kinetic analysis equally effective for both mass and nonmass-like enhancement?. Med. Phys..

[21] Tao W., Hu C., Bai G., Zhu Y., Zhu Y. (2018). Correlation between the dynamic contrast-enhanced MRI features and prognostic factors in breast cancer: A retrospective case-control study. Medicine.

[22] Pereira N.P., Curi C., Osório C.A.B.T., Marques E.F., Makdissi F.B., Pinker K., Bitencourt A.G.V. (2019). Diffusion-Weighted Magnetic Resonance Imaging of Patients with Breast Cancer Following Neoadjuvant Chemotherapy Provides Early Prediction of Pathological Response—A Prospective Study. Sci. Rep..

[23] Narayanan D., Berg W.A. (2018). Dedicated Breast Gamma Camera Imaging and Breast PET: Current Status and Future Directions. PET Clin..

[24] Ferrucci M., Franceschini G., Douek M. (2018). New techniques for sentinel node biopsy in breast cancer. Trans. Cancer Res..

[25] Nandu V.V., Chaudhari M.S. (2017). Efficacy of Sentinel Lymph Node Biopsy in Detecting Axillary Metastasis in Breast Cancer Using Methylene Blue. Indian J. Surg. Oncol..

[26] Brem R.F., Ruda R.C., Yang J.L., Coffey C.M., Rapelyea J.A. (2016). Breast-Specific γ-Imaging for the Detection of Mammographically Occult Breast Cancer in Women at Increased Risk. J. Nucl. Med..

[27] Holbrook A., Newel M.S. (2015). Alternative Screening for Women With Dense Breasts: Breast-Specific Gamma Imaging (Molecular Breast Imaging). Am. J. Roentgenol..

[28] Liu H., Zhan H., Sun D., Zhang Y. (2020). Comparison of BSGI, MRI, mammography, and ultrasound for the diagnosis of breast lesions and their correlations with specific molecular subtypes in Chinese women. BMC Med. Imaging.

[29] Screening for Breast Cancer: U.S. (2016). Preventive Services Task Force Recommendation Statement. Ann. Intern. Med..

[30] Gøtzsche P.C., Jørgensen K.J. (2013). Screening for breast cancer with mammography. Cochrane Database Syst. Rev..

[31] Bhan A. Comparative Analysis of Pre-Processing Techniques for Mammogram Image Enhancement. Proceedings of the INCON VIII 2013: International Conference on Ongoing Research in Management and IT.

[32] 32. Sundaram K.M., Sasikala D., Rani P.A. (2014). A Study On Preprocessing A MammogramImage Using Adaptive Median Filter. Int. J. Innov. Res. Sci. Eng. Technol..

[33] Mandelblatt J.S., Cronin K.A., Bailey S., Berry D.A., de Koning H.J., Draisma G., Huang H., Lee S.J., Munsell M., Plevritis S.K. (2009). Effects of mammography screening under different screening schedules: Model estimates of potential benefits and harms. Ann. Intern. Med..

[34] Nelson H.D., Tyne K., Naik A., Bougatsos C., Chan B.K., Humphrey L. (2009). Screening for breast cancer: An update for the U.S. Preventive Services Task Force. Ann. Intern. Med..

[35] Sprague B.L., Conant E.F., Onega T., Garcia M.P., Beaber E.F., Herschorn S.D., Lehman C.D., Tosteson A.N.A., Lacson R., Schnall M.D. (2016). Variation in Mammographic Breast Density Assessments Among Radiologists in Clinical Practice: A Multicenter Observational Study. Ann. Intern. Med..

[36] Yala A., Lehman C., Schuster T., Portnoi T., Barzilay R. (2019). A Deep Learning Mammography-based Model for Improved Breast Cancer Risk Prediction. Radiology.

[37] Torrisi L., Restuccia N., Torrisi A. (2019). Study of gold nanoparticles for mammography diagnostic and radiotherapy improvements. Rep. Pract. Oncol. Radiother. J. Greatpoland Cancer Cent. Pozn. Pol. Soc. Radiat. Oncol..

[38] Graves M.J., Zhu C., Trivedi R., Saba L., Suri J.S. (2015). Basic Principles of Magnetic Resonance Imaging. 3D Imaging Technologies in Atherosclerosis.

[39] Jethava A., Ali S., Wakefield D., Crowell R., Sporn J., Vrendenburgh J. (2015). Diagnostic Accuracy of MRI in Predicting Breast Tumor Size: Comparative Analysis of MRI vs Histopathological Assessed Breast Tumor Size. Conn. Med..

[40] Grimsby G.M., Gray R., Dueck A., Carpenter S., Stucky C.-C., Aspey H., Giurescu M.E., Pockaj B. (2009). Is there concordance of invasive breast cancer pathologic tumor size with magnetic resonance imaging?. Am. J. Surg..

[41] Goldsmith M., Koutcher J.A., Damadian R. (1978). NMR in cancer, XIII: Application of the NMR malignancy index to human mammary tumours. Br. J. Cancer.

[42] Niell B.L., Gavenonis S.C., Motazedi T., Chubiz J.C., Halpern E.P., Rafferty E.A., Lee J.M. (2014). Auditing a Breast MRI Practice: Performance Measures for Screening and Diagnostic Breast MRI. J. Am. Coll. Radiol..

[43] Michel S.C.A., Keller T.M., Fröhlich J.M., Fink D., Caduff R., Seifert B., Marincek B., Kubik-Huch R.A. (2002). Preoperative Breast Cancer Staging: MR Imaging of the Axilla with Ultrasmall Superparamagnetic Iron Oxide Enhancement. Radiology.

[44] Ayat N.R., Vaidya A., Yeung G.A., Buford M.N., Hall R.C., Qiao P.L., Yu X., Lu Z.-R. (2019). Effective MR Molecular Imaging of Triple Negative Breast Cancer With an EDB-Fibronectin-Specific Contrast Agent at Reduced Doses. Front. Oncol..

[45] Leithner D., Moy L., Morris E.A., Marino M.A., Helbich T.H., Pinker K. (2019). Abbreviated MRI of the Breast: Does It Provide Value?. J. Magn. Reson. Imaging.

[46] Peters N.H.G.M., Borel Rinkes I.H.M., Zuithoff N.P.A., Mali W.P.T.M., Moons K.G.M., Peeters P.H.M. (2008). Meta-analysis of MR imaging in the diagnosis of breast lesions. Radiology.

[47] Sardanelli F., Podo F., D’Agnolo G., Verdecchia A., Santaquilani M., Musumeci R., Trecate G., Manoukian S., Morassut S., de Giacomi C. (2007). Multicenter comparative multimodality surveillance of women at genetic-familial high risk for breast cancer (HIBCRIT study): Interim results. Radiology.

[48] Rahbar H., Partridge S.C. (2016). Multiparametric MR Imaging of Breast Cancer. Magn. Reson. Imaging Clin. North Am..

[49] Teifke A., Behr O., Schmidt M., Victor A., Vomweg T.W., Thelen M., Lehr H.-A. (2006). Dynamic MR imaging of breast lesions: Correlation with microvessel distribution pattern and histologic characteristics of prognosis. Radiology.

[50] Lee S.H., Cho N., Kim S.J., Cha J.H., Cho K.S., Ko E.S., Moon W.K. (2008). Correlation between high resolution dynamic MR features and prognostic factors in breast cancer. Korean J. Radiol..

[51] Choi E.J., Choi H., Choi S.A., Youk J.H. (2016). Dynamic contrast-enhanced breast magnetic resonance imaging for the prediction of early and late recurrences in breast cancer. Medicine.

[52] Wang H., Hu Y., Li H., Xie Y., Wang X., Wan W. (2020). Preliminary study on identification of estrogen receptor-positive breast cancer subtypes based on dynamic contrast-enhanced magnetic resonance imaging (DCE-MRI) texture analysis. Gland Surg..

[53] Gillman J., Toth H.K., Moy L. (2014). The Role of Dynamic Contrast-Enhanced Screening Breast MRI in Populations at Increased Risk for Breast Cancer. Women’s Health.

[54] Turnbull L.W. (2009). Dynamic contrast-enhanced MRI in the diagnosis and management of breast cancer. NMR Biomed..

[55] Amornsiripanitch N., Bickelhaupt S., Shin H.J., Dang M., Rahbar H., Pinker K., Partridge S.C. (2019). Diffusion-weighted MRI for Unenhanced Breast Cancer Screening. Radiology.

[56] Glaser K.J., Manduca A., Ehman R.L. (2012). Review of MR elastography applications and recent developments. J. Magn. Reson. Imaging.

[57] Patel B.K., Samreen N., Zhou Y., Chen J., Brandt K., Ehman R., Pepin K. (2021). MR Elastography of the Breast: Evolution of Technique, Case Examples, and Future Directions. Clin. Breast Cancer.

[58] Hawley J.R., Kalra P., Mo X., Raterman B., Yee L.D., Kolipaka A. (2017). Quantification of breast stiffness using MR elastography at 3 Tesla with a soft sternal driver: A reproducibility study. J. Magn. Reson. Imaging.

[59] McKnight A.L., Kugel J.L., Rossman P.J., Manduca A., Hartmann L.C., Ehman R.L. (2002). MR Elastography of Breast Cancer: Preliminary Results. Am. J. Roentgenol..

[60] Pepin K.M., Ehman R.L., McGee K.P. (2015). Magnetic resonance elastography (MRE) in cancer: Technique, analysis, and applications. Prog. Nucl. Magn. Reson. Spectrosc..

[61] Sinkus R., Siegmann K., Xydeas T., Tanter M., Claussen C., Fink M. (2007). MR elastography of breast lesions: Understanding the solid/liquid duality can improve the specificity of contrast-enhanced MR mammography. Magn. Reson. Med..

[62] Manduca A., Oliphant T.E., Dresner M.A., Mahowald J.L., Kruse S.A., Amromin E., Felmlee J.P., Greenleaf J.F., Ehman R.L. (2001). Magnetic resonance elastography: Non-invasive mapping of tissue elasticity. Med. Image Anal..

[63] Lorenzen J., Sinkus R., Lorenzen M., Dargatz M., Leussler C., Röschmann P., Adam G. (2002). MR elastography of the breast:preliminary clinical results. Rofo Fortschr. Geb. Rontgenstrahlen Nukl..

[64] Malayeri A.A., El Khouli R.H., Zaheer A., Jacobs M.A., Corona-Villalobos C.P., Kamel I.R., Macura K.J. (2011). Principles and Applications of Diffusion-weighted Imaging in Cancer Detection, Staging, and Treatment Follow-up. RadioGraphics.

[65] Petralia G., Bonello L., Priolo F., Summers P., Bellomi M. (2011). Breast MR with special focus on DW-MRI and DCE-MRI. Cancer Imaging Off. Publ. Int. Cancer Imaging Soc..

[66] Baron P., Dorrius M.D., Kappert P., Oudkerk M., Sijens P.E. (2010). Diffusion-weighted imaging of normal fibroglandular breast tissue: Influence of microperfusion and fat suppression technique on the apparent diffusion coefficient. NMR Biomed..

[67] Durur-Subasi I. (2019). DW-MRI of the breast: A pictorial review. Insights Into Imaging.

[68] Baltzer P.A.T., Kapetas P., Sodano C., Dietzel M., Pinker K., Helbich T.H., Clauser P. (2019). Contrast agent-free breast MRI: Advantages and potential disadvantages. Der Radiol..

[69] Baltzer P., Mann R.M., Iima M., Sigmund E.E., Clauser P., Gilbert F.J., Martincich L., Partridge S.C., Patterson A., Pinker K. (2020). Diffusion-weighted imaging of the breast-a consensus and mission statement from the EUSOBI International Breast Diffusion-Weighted Imaging working group. Eur. Radiol..

[70] Bolan P.J., Meisamy S., Baker E.H., Lin J., Emory T., Nelson M., Everson L.I., Yee D., Garwood M. (2003). In vivo quantification of choline compounds in the breast with 1H MR spectroscopy. Magn. Reson. Med..

[71] Jagannathan N.R., Kumar M., Seenu V., Coshic O., Dwivedi S.N., Julka P.K., Srivastava A., Rath G.K. (2001). Evaluation of total choline from in-vivo volume localized proton MR spectroscopy and its response to neoadjuvant chemotherapy in locally advanced breast cancer. Br. J. Cancer.

[72] Meisamy S., Bolan P.J., Baker E.H., Pollema M.G., Le C.T., Kelcz F., Lechner M.C., Luikens B.A., Carlson R.A., Brandt K.R. (2005). Adding in Vivo Quantitative 1H MR Spectroscopy to Improve Diagnostic Accuracy of Breast MR Imaging: Preliminary Results of Observer Performance Study at 4.0 T. Radiology.

[73] Stanwell P., Mountford C. (2007). In Vivo Proton MR Spectroscopy of the Breast. RadioGraphics.

[74] Liberti M.V., Locasale J.W. (2016). The Warburg Effect: How Does it Benefit Cancer Cells?. Trends Biochem. Sci..

[75] Flavell R.R., Naeger D.M., Mari Aparici C., Hawkins R.A., Pampaloni M.H., Behr S.C. (2016). Malignancies with Low Fluorodeoxyglucose Uptake at PET/CT: Pitfalls and Prognostic Importance: Resident and Fellow Education Feature. RadioGraphics.

[76] Kawada K., Iwamoto M., Sakai Y. (2016). Mechanisms underlying (18)F-fluorodeoxyglucose accumulation in colorectal cancer. World J. Radiol..

[77] Kadoya T., Aogi K., Kiyoto S., Masumoto N., Sugawara Y., Okada M. (2013). Role of maximum standardized uptake value in fluorodeoxyglucose positron emission tomography/computed tomography predicts malignancy grade and prognosis of operable breast cancer: A multi-institute study. Breast Cancer Res. Treat..

[78] Jin S., Kim S.-B., Ahn J.-H., Jung K.H., Ahn S.H., Son B.H., Lee J.W., Gong G., Kim H.O., Moon D.H. (2013). 18 F-fluorodeoxyglucose uptake predicts pathological complete response after neoadjuvant chemotherapy for breast cancer: A retrospective cohort study. J. Surg. Oncol..

[79] Narayanan D., Madsen K.S., Kalinyak J.E., Berg W.A. (2011). Interpretation of Positron Emission Mammography and MRI by Experienced Breast Imaging Radiologists: Performance and Observer Reproducibility. Am. J. Roentgenol..

[80] Hsu D.F.C., Freese D.L., Levin C.S. (2016). Breast-Dedicated Radionuclide Imaging Systems. J. Nucl. Med..

[81] Giammarile F., Castellucci P., Dierckx R., Estrada Lobato E., Farsad M., Hustinx R., Jalilian A., Pellet O., Rossi S., Paez D. (2019). Non-FDG PET/CT in Diagnostic Oncology: A pictorial review. Eur. J. Hybrid Imaging.

[82] Blanchet E.M., Millo C., Martucci V., Maass-Moreno R., Bluemke D.A., Pacak K. (2014). Integrated whole-body PET/MRI with 18F-FDG, 18F-FDOPA, and 18F-FDA in paragangliomas in comparison with PET/CT: NIH first clinical experience with a single-injection, dual-modality imaging protocol. Clin. Nucl. Med..

[83] Wibmer A.G., Hricak H., Ulaner G.A., Weber W. (2018). Trends in oncologic hybrid imaging. Eur. J. Hybrid Imaging.

[84] Escalona S., Blasco J.A., Reza M.M., Andradas E., Gómez N. (2010). A systematic review of FDG-PET in breast cancer. Med. Oncol..

[85] Jørgensen J.T., Norregaard K., Simón Martín M., Oddershede L.B., Kjaer A. (2018). Non-invasive Early Response Monitoring of Nanoparticle-assisted Photothermal Cancer Therapy Using (18)F-FDG, (18)F-FLT, and (18)F-FET PET/CT Imaging. Nanotheranostics.

[86] Lyman G.H., Somerfield M.R., Bosserman L.D., Perkins C.L., Weaver D.L., Giuliano A.E. (2016). Sentinel Lymph Node Biopsy for Patients With Early-Stage Breast Cancer: American Society of Clinical Oncology Clinical Practice Guideline Update. J. Clin. Oncol..

[87] Cox C.E., Kiluk J.V., Riker A.I., Cox J.M., Allred N., Ramos D.C., Dupont E.L., Vrcel V., Diaz N., Boulware D. (2008). Significance of sentinel lymph node micrometastases in human breast cancer. J. Am. Coll. Surg..

[88] Chen S.L., Iddings D.M., Scheri R.P., Bilchik A.J. (2006). Lymphatic mapping and sentinel node analysis: Current concepts and applications. CA A Cancer J. Clin..

[89] Kang J., Chang J.H., Kim S.M., Lee H.J., Kim H., Wilson B.C., Song T.-K. (2017). Real-time sentinel lymph node biopsy guidance using combined ultrasound, photoacoustic, fluorescence imaging: In vivo proof-of-principle and validation with nodal obstruction. Sci. Rep..

[90] Shaikh K., Krishnan S., Thanki R. (2021). Breast Cancer Detection and Diagnosis Using AI. Artificial Intelligence in Breast Cancer Early Detection and Diagnosis.

[91] Liu H., Zhan H., Sun D. (2020). Comparison of 99mTc-MIBI scintigraphy, ultrasound, and mammography for the diagnosis of BI-RADS 4 category lesions. BMC Cancer.

[92] Gong Z., Williams M.B. (2015). Comparison of breast specific gamma imaging and molecular breast tomosynthesis in breast cancer detection: Evaluation in phantoms. Med. Phys..

[93] Rechtman L.R., Lenihan M.J., Lieberman J.H., Teal C.B., Torrente J., Rapelyea J.A., Brem R.F. (2014). Breast-Specific Gamma Imaging for the Detection of Breast Cancer in Dense Versus Nondense Breasts. Am. J. Roentgenol..

[94] Zhang Z., Wang W., Wang X., Yu X., Zhu Y., Zhan H., Chen Z., Li B., Huang J. (2020). Breast-specific gamma imaging or ultrasonography as adjunct imaging diagnostics in women with mammographically dense breasts. Eur. Radiol..

[95] Surti S. (2013). Radionuclide methods and instrumentation for breast cancer detection and diagnosis. Semin. Nucl. Med..

[96] Urbano N., Scimeca M., Tancredi V., Bonanno E., Schillaci O. (2020). 99mTC-sestamibi breast imaging: Current status, new ideas and future perspectives. Semin. Cancer Biol..

[97] Candelaria R., Fornage B.D. (2011). Second-look US examination of MR-detected breast lesions. J. Clin. Ultrasound.

[98] Sridharan A., Eisenbrey J.R., Machado P., Ojeda-Fournier H., Wilkes A., Sevrukov A., Mattrey R.F., Wallace K., Chalek C.L., Thomenius K.E. (2015). Quantitative analysis of vascular heterogeneity in breast lesions using contrast-enhanced 3-D harmonic and subharmonic ultrasound imaging. IEEE Trans. Ultrason. Ferroelectr. Freq. Control.

[99] Kaplan S.S. (2014). Automated whole breast ultrasound. Radiol. Clin. North Am..

[100] Skerl K., Vinnicombe S., Thomson K., McLean D., Giannotti E., Evans A. (2016). Anisotropy of Solid Breast Lesions in 2D Shear Wave Elastography is an Indicator of Malignancy. Acad. Radiol..

[101] Bamber J., Cosgrove D., Dietrich C.F., Fromageau J., Bojunga J., Calliada F., Cantisani V., Correas J.-M., D’Onofrio M., Drakonaki E.E. (2013). EFSUMB guidelines and recommendations on the clinical use of ultrasound elastography. Part 1: Basic principles and technology. Ultraschall in der Medizin.

[102] Wan C.F., Du J., Fang H., Li F.H., Zhu J.S., Liu Q. (2012). Enhancement patterns and parameters of breast cancers at contrast-enhanced US: Correlation with prognostic factors. Radiology.

[103] Gill M.R., Menon J.U., Jarman P.J., Owen J., Skaripa-Koukelli I., Able S., Thomas J.A., Carlisle R., Vallis K.A. (2018). (111)In-labelled polymeric nanoparticles incorporating a ruthenium-based radiosensitizer for EGFR-targeted combination therapy in oesophageal cancer cells. Nanoscale.

[104] Sood R., Rositch A.F., Shakoor D., Ambinder E., Pool K.-L., Pollack E., Mollura D.J., Mullen L.A., Harvey S.C. (2019). Ultrasound for Breast Cancer Detection Globally: A Systematic Review and Meta-Analysis. J. Glob. Oncol..

[105] Polyak K. (2011). Heterogeneity in breast cancer. J. Clin. Investig..

[106] Hammond M.E.H., Hayes D.F., Dowsett M., Allred D.C., Hagerty K.L., Badve S., Fitzgibbons P.L., Francis G., Goldstein N.S., Hayes M. (2010). American Society of Clinical Oncology/College of American Pathologists Guideline Recommendations for Immunohistochemical Testing of Estrogen and Progesterone Receptors in Breast Cancer. J. Clin. Oncol..

[107] Wolff A.C., Hammond M.E.H., Hicks D.G., Dowsett M., McShane L.M., Allison K.H., Allred D.C., Bartlett J.M.S., Bilous M., Fitzgibbons P. (2014). Recommendations for human epidermal growth factor receptor 2 testing in breast cancer: American Society of Clinical Oncology/College of American Pathologists clinical practice guideline update. Arch. Pathol. Lab. Med..

[108] Yanagawa M., Ikemot K., Kawauchi S., Furuya T., Yamamoto S., Oka M., Oga A., Nagashima Y., Sasaki K. (2012). Luminal A and luminal B (HER2 negative) subtypes of breast cancer consist of a mixture of tumors with different genotype. BMC Res. Notes.

[109] Prado-Vázquez G., Gámez-Pozo A., Trilla-Fuertes L., Arevalillo J.M., Zapater-Moros A., Ferrer-Gómez M., Díaz-Almirón M., López-Vacas R., Navarro H., Maín P. (2019). A novel approach to triple-negative breast cancer molecular classification reveals a luminal immune-positive subgroup with good prognoses. Sci. Rep..

[110] Wang J., Xu B. (2019). Targeted therapeutic options and future perspectives for HER2-positive breast cancer. Signal Transduct. Target. Ther..

[111] Love R.R., Koroltchouk V. (1993). Tamoxifen therapy in breast cancer control worldwide. Bull. World Health Organ..

[112] Meiser B., Wong W.K.T., Peate M., Julian-Reynier C., Kirk J., Mitchell G. (2017). Motivators and barriers of tamoxifen use as risk-reducing medication amongst women at increased breast cancer risk: A systematic literature review. Hered. Cancer Clin. Pract..

[113] Cuzick J., Sestak I., Bonanni B., Costantino J.P., Cummings S., DeCensi A., Dowsett M., Forbes J.F., Ford L., LaCroix A.Z. (2013). Selective oestrogen receptor modulators in prevention of breast cancer: An updated meta-analysis of individual participant data. Lancet.

[114] Nazarali S.A., Narod S.A. (2014). Tamoxifen for women at high risk of breast cancer. Breast Cancer.

[115] Narod S.A. (2015). Tamoxifen Chemoprevention—End of the Road?. JAMA Oncol..

[116] Fabian C.J. (2007). The what, why and how of aromatase inhibitors: Hormonal agents for treatment and prevention of breast cancer. Int. J. Clin. Pract..

[117] Schneider R., Barakat A., Pippen J., Osborne C. (2011). Aromatase inhibitors in the treatment of breast cancer in post-menopausal female patients: An update. Breast Cancer.

[118] (2002). Anastrozole Approved for HR-Positive Early Breast Cancer. Oncol. Times.

[119] Cohen M.H., Johnson J.R., Li N., Chen G., Pazdur R. (2002). Approval summary: Letrozole in the treatment of postmenopausal women with advanced breast cancer. Clin. Cancer Res. Off. J. Am. Assoc. Cancer Res..

[120] (2005). FDA Approval for Exemestane for Adjuvant Treatment for Early Breast Cancer in Postmenopausal Women. Oncol. Times.

[121] Tan S.-H., Wolff A.C. (2007). Luteinizing Hormone-Releasing Hormone Agonists in Premenopausal Hormone Receptor–Positive Breast Cancer. Clin. Breast Cancer.

[122] Rocca A., Maltoni R., Bravaccini S., Donati C., Andreis D. (2018). Clinical utility of fulvestrant in the treatment of breast cancer: A report on the emerging clinical evidence. Cancer Manag. Res..

[123] Wakeling A.E., Dukes M., Bowler J. (1991). A potent specific pure antiestrogen with clinical potential. Cancer Res..

[124] Xu H., Yu S., Liu Q., Yuan X., Mani S., Pestell R.G., Wu K. (2017). Recent advances of highly selective CDK4/6 inhibitors in breast cancer. J. Hematol. Oncol..

[125] Niu Y., Xu J., Sun T. (2019). Cyclin-Dependent Kinases 4/6 Inhibitors in Breast Cancer: Current Status, Resistance, and Combination Strategies. J. Cancer.

[126] Li X., Dai D., Chen B., Tang H., Xie X., Wei W. (2018). Efficacy of PI3K/AKT/mTOR pathway inhibitors for the treatment of advanced solid cancers: A literature-based meta-analysis of 46 randomised control trials. PLoS ONE.

[127] Krop I.E., Mayer I.A., Ganju V., Dickler M., Johnston S., Morales S., Yardley D.A., Melichar B., Forero-Torres A., Lee S.C. (2016). Pictilisib for oestrogen receptor-positive, aromatase inhibitor-resistant, advanced or metastatic breast cancer (FERGI): A randomised, double-blind, placebo-controlled, phase 2 trial. Lancet Oncol..

[128] Rodon J., Braña I., Siu L.L., De Jonge M.J., Homji N., Mills D., Di Tomaso E., Sarr C., Trandafir L., Massacesi C. (2014). Phase I dose-escalation and -expansion study of buparlisib (BKM120), an oral pan-Class I PI3K inhibitor, in patients with advanced solid tumors. Investig. New Drugs.

[129] Folkes A.J., Ahmadi K., Alderton W.K., Alix S., Baker S.J., Box G., Chuckowree I.S., Clarke P.A., Depledge P., Eccles S.A. (2008). The identification of 2-(1H-indazol-4-yl)-6-(4-methanesulfonyl-piperazin-1-ylmethyl)-4-morpholin-4-yl-thieno[3,2-d]pyrimidine (GDC-0941) as a potent, selective, orally bioavailable inhibitor of class I PI3 kinase for the treatment of cancer. J. Med. Chem..

[130] Sarker D., Ang J.E., Baird R., Kristeleit R., Shah K., Moreno V., Clarke P.A., Raynaud F.I., Levy G., Ware J.A. (2015). First-in-Human Phase I Study of Pictilisib (GDC-0941), a Potent Pan{\textendash}Class I Phosphatidylinositol-3-Kinase (PI3K) Inhibitor, in Patients with Advanced Solid Tumors. Clin. Cancer Res..

[131] Shapiro G.I., Rodon J., Bedell C., Kwak E.L., Baselga J., Braña I., Pandya S.S., Scheffold C., Laird A.D., Nguyen L.T. (2014). Phase I Safety, Pharmacokinetic, and Pharmacodynamic Study of SAR245408 (XL147), an Oral Pan-Class I PI3K Inhibitor, in Patients with Advanced Solid Tumors. Clin. Cancer Res..

[132] Blackwell K., Burris H., Gomez P., Lynn Henry N., Isakoff S., Campana F., Gao L., Jiang J., Macé S., Tolaney S.M. (2015). Phase I/II dose-escalation study of PI3K inhibitors pilaralisib or voxtalisib in combination with letrozole in patients with hormone-receptor-positive and HER2-negative metastatic breast cancer refractory to a non-steroidal aromatase inhibitor. Breast Cancer Res. Treat..

[133] Albanell J., Baselga J. (1999). Trastuzumab, a humanized anti-HER2 monoclonal antibody, for the treatment of breast cancer. Drugs Today.

[134] Saura C., Bendell J., Jerusalem G., Su S., Ru Q., De Buck S., Mills D., Ruquet S., Bosch A., Urruticoechea A. (2014). Phase Ib study of Buparlisib plus Trastuzumab in patients with HER2-positive advanced or metastatic breast cancer that has progressed on Trastuzumab-based therapy. Clin. Cancer Res. Off. J. Am. Assoc. Cancer Res..

[135] Tolaney S., Burris H., Gartner E., Mayer I.A., Saura C., Maurer M., Ciruelos E., Garcia A.A., Campana F., Wu B. (2015). Phase I/II study of pilaralisib (SAR245408) in combination with trastuzumab or trastuzumab plus paclitaxel in trastuzumab-refractory HER2-positive metastatic breast cancer. Breast Cancer Res. Treat..

[136] Ishii K., Morii N., Yamashiro H. (2019). Pertuzumab in the treatment of HER2-positive breast cancer: An evidence-based review of its safety, efficacy, and place in therapy. Core Evid..

[137] Chan A., Delaloge S., Holmes F.A., Moy B., Iwata H., Harvey V.J., Robert N.J., Silovski T., Gokmen E., von Minckwitz G. (2016). Neratinib after trastuzumab-based adjuvant therapy in patients with HER2-positive breast cancer (ExteNET): A multicentre, randomised, double-blind, placebo-controlled, phase 3 trial. Lancet Oncol..

[138] Mukai H., Saeki T., Aogi K., Naito Y., Matsubara N., Shigekawa T., Ueda S., Takashima S., Hara F., Yamashita T. (2016). Patritumab plus trastuzumab and paclitaxel in human epidermal growth factor receptor 2-overexpressing metastatic breast cancer. Cancer Sci..

[139] Earl H.M., Hiller L., Dunn J.A., Blenkinsop C., Grybowicz L., Vallier A.-L., Abraham J., Thomas J., Provenzano E., Hughes-Davies L. (2015). Efficacy of neoadjuvant bevacizumab added to docetaxel followed by fluorouracil, epirubicin, and cyclophosphamide, for women with HER2-negative early breast cancer (ARTemis): An open-label, randomised, phase 3 trial. Lancet Oncol..

[140] Verret B., Cortes J., Bachelot T., Andre F., Arnedos M. (2019). Efficacy of PI3K inhibitors in advanced breast cancer. Ann. Oncol. Off. J. Eur. Soc. Med. Oncol..

[141] Peddi P.F., Hurvitz S.A. (2014). Ado-trastuzumab emtansine (T-DM1) in human epidermal growth factor receptor 2 (HER2)-positive metastatic breast cancer: Latest evidence and clinical potential. Ther. Adv. Med. Oncol..

[142] Modi S., Saura C., Yamashita T., Park Y.H., Kim S.-B., Tamura K., Andre F., Iwata H., Ito Y., Tsurutani J. (2020). Trastuzumab Deruxtecan in Previously Treated HER2-Positive Breast Cancer. N. Engl. J. Med..

[143] Kim R., Keam B., Hahn S., Ock C.-Y., Kim M., Kim T.M., Kim D.-W., Heo D.S. (2019). First-line Pembrolizumab versus Pembrolizumab Plus Chemotherapy Versus Chemotherapy Alone in Non-small-cell Lung Cancer: A Systematic Review and Network Meta-analysis. Clin. Lung Cancer.

[144] Kang C., Syed Y.Y. (2020). Atezolizumab (in Combination with Nab-Paclitaxel): A Review in Advanced Triple-Negative Breast Cancer. Drugs.

[145] Huerta-Reyes M., Maya-Núñez G., Pérez-Solis M.A., López-Muñoz E., Guillén N., Olivo-Marin J.-C., Aguilar-Rojas A. (2019). Treatment of Breast Cancer With Gonadotropin-Releasing Hormone Analogs. Front. Oncol..

[146] Schally A.V., Engel J.B., Pinski J., Block N.L. (2013). Chapter 73—LHRH Analogs. Handbook of Biologically Active Peptides.

[147] Weir H.M., Bradbury R.H., Lawson M., Rabow A.A., Buttar D., Callis R.J., Curwen J.O., de Almeida C., Ballard P., Hulse M. (2016). AZD9496: An oral estrogen receptor inhibitor that blocks the growth of ER-positive and ESR1-mutant breast tumors in preclinical models. Cancer Res..

[148] Xu S., Yu S., Dong D., Lee L.T.O. (2019). G Protein-Coupled Estrogen Receptor: A Potential Therapeutic Target in Cancer. Front. Endocrinol..

[149] Lykkesfeldt A.E., Larsen S.S., Briand P. (1995). Human breast cancer cell lines resistant to pure anti-estrogens are sensitive to tamoxifen treatment. Int. J. Cancer.

[150] Osborne C.K., Coronado-Heinsohn E.B., Hilsenbeck S.G., McCue B.L., Wakeling A.E., McClelland R.A., Manning D.L., Nicholson R.I. (1995). Comparison of the effects of a pure steroidal antiestrogen with those of tamoxifen in a model of human breast cancer. J. Natl. Cancer Inst..

[151] Dowsett M., Nicholson R.I., Pietras R.J. (2005). Biological characteristics of the pure antiestrogen fulvestrant: Overcoming endocrine resistance. Breast Cancer Res. Treat..

[152] Michaud L.B., Jones K.L., Buzdar A.U. (2001). Combination endocrine therapy in the management of breast cancer. Oncologist.

[153] Bergh J., Jönsson P.-E., Lidbrink E.K., Trudeau M., Eiermann W., Brattström D., Lindemann J.P.O., Wiklund F., Henriksson R. (2012). FACT: An open-label randomized phase III study of fulvestrant and anastrozole in combination compared with anastrozole alone as first-line therapy for patients with receptor-positive postmenopausal breast cancer. J. Clin. Oncol. Off. J. Am. Soc. Clin. Oncol..

[154] Mehta R.S., Barlow W.E., Albain K.S., Vandenberg T.A., Dakhil S.R., Tirumali N.R., Lew D.L., Hayes D.F., Gralow J.R., Livingston R.B. (2012). Combination Anastrozole and Fulvestrant in Metastatic Breast Cancer. N. Engl. J. Med..

[155] Johnston S.R., Kilburn L.S., Ellis P., Dodwell D., Cameron D., Hayward L., Im Y.-H., Braybrooke J.P., Brunt A.M., Cheung K.-L. (2013). Fulvestrant plus anastrozole or placebo versus exemestane alone after progression on non-steroidal aromatase inhibitors in postmenopausal patients with hormone-receptor-positive locally advanced or metastatic breast cancer (SoFEA): A composite, multicent. Lancet Oncol..

[156] Chen X., Xu D., Li X., Zhang J., Xu W., Hou J., Zhang W., Tang J. (2019). Latest Overview of the Cyclin-Dependent Kinases 4/6 Inhibitors in Breast Cancer: The Past, the Present and the Future. J. Cancer.

[157] Tan A.R., Yang X., Berman A., Zhai S., Sparreboom A., Parr A.L., Chow C., Brahim J.S., Steinberg S.M., Figg W.D. (2004). Phase I Trial of the Cyclin-Dependent Kinase Inhibitor Flavopiridol in Combination with Docetaxel in Patients with Metastatic Breast Cancer. Clin. Cancer Res..

[158] Barroso-Sousa R., Shapiro G.I., Tolaney S.M. (2016). Clinical Development of the CDK4/6 Inhibitors Ribociclib and Abemaciclib in Breast Cancer. Breast Care.

[159] Cristofanilli M., Turner N.C., Bondarenko I., Ro J., Im S.-A., Masuda N., Colleoni M., DeMichele A., Loi S., Verma S. (2016). Fulvestrant plus palbociclib versus fulvestrant plus placebo for treatment of hormone-receptor-positive, HER2-negative metastatic breast cancer that progressed on previous endocrine therapy (PALOMA-3): Final analysis of the multicentre, double-blind, pha. Lancet Oncol..

[160] Hortobagyi G.N., Stemmer S.M., Burris H.A., Yap Y.-S., Sonke G.S., Paluch-Shimon S., Campone M., Blackwell K.L., André F., Winer E.P. (2016). Ribociclib as First-Line Therapy for HR-Positive, Advanced Breast Cancer. N. Engl. J. Med..

[161] Finn R.S., Martin M., Rugo H.S., Jones S., Im S.-A., Gelmon K., Harbeck N., Lipatov O.N., Walshe J.M., Moulder S. (2016). Palbociclib and Letrozole in Advanced Breast Cancer. N. Engl. J. Med..

[162] Liu P., Cheng H., Roberts T.M., Zhao J.J. (2009). Targeting the phosphoinositide 3-kinase pathway in cancer. Nat. Rev. Drug Discov..

[163] Beser A.R., Tuzlali S., Guzey D., Dolek Guler S., Hacihanefioglu S., Dalay N. (2007). HER-2, TOP2A and chromosome 17 alterations in breast cancer. Pathol. Oncol. Res..

[164] Yarden Y. (2001). Biology of HER2 and its importance in breast cancer. Oncology.

[165] Witton C.J., Reeves J.R., Going J.J., Cooke T.G., Bartlett J.M.S. (2003). Expression of the HER1-4 family of receptor tyrosine kinases in breast cancer. J. Pathol..

[166] Cismowski M. (2007). Tyrosine Kinase Inhibitors. xPharm: The Comprehensive Pharmacology Reference.

[167] Xuhong J.-C., Qi X.-W., Zhang Y., Jiang J. (2019). Mechanism, safety and efficacy of three tyrosine kinase inhibitors lapatinib, neratinib and pyrotinib in HER2-positive breast cancer. Am. J. Cancer Res..

[168] Guan Z., Xu B., DeSilvio M.L., Shen Z., Arpornwirat W., Tong Z., Lorvidhaya V., Jiang Z., Yang J., Makhson A. (2013). Randomized trial of lapatinib versus placebo added to paclitaxel in the treatment of human epidermal growth factor receptor 2-overexpressing metastatic breast cancer. J. Clin. Oncol. Off. J. Am. Soc. Clin. Oncol..

[169] Chien A.J., Rugo H.S. (2017). Tyrosine Kinase Inhibitors for Human Epidermal Growth Factor Receptor 2–Positive Metastatic Breast Cancer: Is Personalizing Therapy Within Reach?. J. Clin. Oncol..

[170] Li X., Yang C., Wan H., Zhang G., Feng J., Zhang L., Chen X., Zhong D., Lou L., Tao W. (2017). Discovery and development of pyrotinib: A novel irreversible EGFR/HER2 dual tyrosine kinase inhibitor with favorable safety profiles for the treatment of breast cancer. Eur. J. Pharm. Sci. Off. J. Eur. Fed. Pharm. Sci..

[171] Hudis C.A. (2007). Trastuzumab—Mechanism of Action and Use in Clinical Practice. N. Engl. J. Med..

[172] Adamczyk A., Niemiec J., Janecka A., Harazin-Lechowska A., Ambicka A., Grela-Wojewoda A., Domagała-Haduch M., Cedrych I., Majchrzyk K., Kruczak A. (2015). Prognostic value of PIK3CA mutation status, PTEN and androgen receptor expression for metastasis-free survival in HER2-positive breast cancer patients treated with trastuzumab in adjuvant setting. Pol. J. Pathol. Off. J. Pol. Soc. Pathol..

[173] Loibl S., Gianni L. (2017). HER2-positive breast cancer. Lancet.

[174] Luo C., Zhong X., Wang Z., Wang Y., Wang Y., He P., Peng Q., Zheng H. (2019). Prognostic nomogram for patients with non-metastatic HER2 positive breast cancer in a prospective cohort. Int. J. Biol. Mark..

[175] Adams C.W., Allison D.E., Flagella K., Presta L., Clarke J., Dybdal N., McKeever K., Sliwkowski M.X. (2006). Humanization of a recombinant monoclonal antibody to produce a therapeutic HER dimerization inhibitor, pertuzumab. Cancer Immunol. Immunother..

[176] Franklin M.C., Carey K.D., Vajdos F.F., Leahy D.J., de Vos A.M., Sliwkowski M.X. (2004). Insights into ErbB signaling from the structure of the ErbB2-pertuzumab complex. Cancer Cell.

[177] Dawson S.J., Provenzano E., Caldas C. (2009). Triple negative breast cancers: Clinical and prognostic implications. Eur. J. Cancer.

[178] Anders C., Carey L.A. (2008). Understanding and treating triple-negative breast cancer. Oncology.

[179] Liedtke C., Mazouni C., Hess K.R., André F., Tordai A., Mejia J.A., Symmans W.F., Gonzalez-Angulo A.M., Hennessy B., Green M. (2008). Response to neoadjuvant therapy and long-term survival in patients with triple-negative breast cancer. J. Clin. Oncol. Off. J. Am. Soc. Clin. Oncol..

[180] Lin N.U., Vanderplas A., Hughes M.E., Theriault R.L., Edge S.B., Wong Y.-N., Blayney D.W., Niland J.C., Winer E.P., Weeks J.C. (2012). Clinicopathologic features, patterns of recurrence, and survival among women with triple-negative breast cancer in the National Comprehensive Cancer Network. Cancer.

[181] Tong C.W.S., Wu M., Cho W.C.S., To K.K.W. (2018). Recent Advances in the Treatment of Breast Cancer. Front. Oncol..

[182] Tutt A., Tovey H., Cheang M.C.U., Kernaghan S., Kilburn L., Gazinska P., Owen J., Abraham J., Barrett S., Barrett-Lee P. (2018). Carboplatin in BRCA1/2-mutated and triple-negative breast cancer BRCAness subgroups: The TNT Trial. Nat. Med..

[183] Bardia A., Mayer I., Vahdat L., Tolaney S., Isakoff S., Diamond J., O’Shaughnessy J., Moroose R., Santin A., Abramson V. (2019). Sacituzumab Govitecan-hziy in Refractory Metastatic Triple-Negative Breast Cancer. N. Engl. J. Med..

[184] Aschenbrenner D.S. (2020). New Drug Approved for HER2-positive Metastatic Breast Cancer. Am. J. Nurs..

[185] Jeelani S., Reddy R.C.J., Maheswaran T., Asokan G.S., Dany A., Anand B. (2014). Theranostics: A treasured tailor for tomorrow. J. Pharm. Bioallied Sci..

[186] Shah J.V., Gonda A., Pemmaraju R., Subash A., Bobadilla Mendez C., Berger M., Zhao X., He S., Riman R.E., Tan M.C. (2020). Shortwave Infrared-Emitting Theranostics for Breast Cancer Therapy Response Monitoring. Front. Mol. Biosci..

[187] Bartelink I.H., Jones E.F., Shahidi-Latham S.K., Lee P.R.E., Zheng Y., Vicini P., van ’t Veer L., Wolf D., Iagaru A., Kroetz D.L. (2019). Tumor Drug Penetration Measurements Could Be the Neglected Piece of the Personalized Cancer Treatment Puzzle. Clin. Pharmacol. Ther..

[188] Tannock I.F. (1998). Conventional cancer therapy: Promise broken or promise delayed?. Lancet.

[189] Ahn B.-C. (2016). Personalized Medicine Based on Theranostic Radioiodine Molecular Imaging for Differentiated Thyroid Cancer. Biomed. Res. Int..

[190] Thakur V., Kutty R.V. (2019). Recent advances in nanotheranostics for triple negative breast cancer treatment. J. Exp. Clin. Cancer Res..

[191] Engebraaten O., Vollan H.K.M., Børresen-Dale A.-L. (2013). Triple-negative breast cancer and the need for new therapeutic targets. Am. J. Pathol..

[192] Sumer B., Gao J. (2008). Theranostic nanomedicine for cancer. Nanomedicine.

[193] Gregoriou Y., Gregoriou G., Yilmaz V., Kapnisis K., Prokopi M., Anayiotos A., Strati K., Dietis N., Constantinou A.I., Andreou C. (2020). Resveratrol loaded polymeric micelles for theranostic targeting of breast cancer cells. Nanotheranostics.

[194] Wang Y., Wang Y., Chen G., Li Y., Xu W., Gong S. (2017). Quantum-Dot-Based Theranostic Micelles Conjugated with an Anti-EGFR Nanobody for Triple-Negative Breast Cancer Therapy. ACS Appl. Mater. Interfaces.

[195] Parhi P., Sahoo S.K. (2015). Trastuzumab guided nanotheranostics: A lipid based multifunctional nanoformulation for targeted drug delivery and imaging in breast cancer therapy. J. Colloid Interface Sci..

[196] Hafner S., Raabe M., Wu Y., Wang T., Zuo Z., Rasche V., Syrovets T., Weil T., Simmet T. (2019). High-Contrast Magnetic Resonance Imaging and Efficient Delivery of an Albumin Nanotheranostic in Triple-Negative Breast Cancer Xenografts. Adv. Ther..

[197] Li J., Cai P., Shalviri A., Henderson J.T., He C., Foltz W.D., Prasad P., Brodersen P.M., Chen Y., DaCosta R. (2014). A Multifunctional Polymeric Nanotheranostic System Delivers Doxorubicin and Imaging Agents across the Blood–Brain Barrier Targeting Brain Metastases of Breast Cancer. ACS Nano.

[198] Li L., Fu J., Wang X., Chen Q., Zhang W., Cao Y., Ran H. (2021). Biomimetic “Nanoplatelets” as a Targeted Drug Delivery Platform for Breast Cancer Theranostics. ACS Appl. Mater. Interfaces.

[199] Dong Q., Yang H., Wan C., Zheng D., Zhou Z., Xie S., Xu L., Du J., Li F. (2019). Her2-Functionalized Gold-Nanoshelled Magnetic Hybrid Nanoparticles: A Theranostic Agent for Dual-Modal Imaging and Photothermal Therapy of Breast Cancer. Nanoscale Res. Lett..

[200] Brabec V., Nováková O. (2006). DNA binding mode of ruthenium complexes and relationship to tumor cell toxicity. Coordination Chem. Rev..

[201] Tan L., Shen J., Liu J., Zeng L., Jin L., Weng C. (2012). Spectral characteristics, DNA-binding and cytotoxicity of two functional Ru (II) mixed-ligand complexes. Dalton Trans..

[202] Shen J., Kim H.-C., Wolfram J., Mu C., Zhang W., Liu H., Xie Y., Mai J., Zhang H., Li Z. (2017). A Liposome Encapsulated Ruthenium Polypyridine Complex as a Theranostic Platform for Triple-Negative Breast Cancer. Nano Lett..

[203] Zheng D., Wan C., Yang H., Xu L., Dong Q., Du C., Du J., Li F. (2020). Her2-Targeted Multifunctional Nano-Theranostic Platform Mediates Tumor Microenvironment Remodeling and Immune Activation for Breast Cancer Treatment. Int. J. Nanomed..

[204] Tang L., Yang X., Yin Q., Cai K., Wang H., Chaudhury I., Yao C., Zhou Q., Kwon M., Hartman J.A. (2014). Investigating the optimal size of anticancer nanomedicine. Proc. Natl. Acad. Sci. USA.

[205] Liu R., Xiao W., Hu C., Xie R., Gao H. (2018). Theranostic size-reducible and no donor conjugated gold nanocluster fabricated hyaluronic acid nanoparticle with optimal size for combinational treatment of breast cancer and lung metastasis. J. Control. Release Off. J. Control. Release Soc..

[206] Liu R., Hu C., Yang Y., Zhang J., Gao H. (2019). Theranostic nanoparticles with tumor-specific enzyme-triggered size reduction and drug release to perform photothermal therapy for breast cancer treatment. Acta Pharm. Sin. B.

[207] Burke A.R., Singh R.N., Carroll D.L., Wood J.C.S., D’Agostino R.B., Ajayan P.M., Torti F.M., Torti S.V. (2012). The resistance of breast cancer stem cells to conventional hyperthermia and their sensitivity to nanoparticle-mediated photothermal therapy. Biomaterials.

[208] Wu Y., Wang H., Gao F., Xu Z., Dai F., Liu W. (2018). An Injectable Supramolecular Polymer Nanocomposite Hydrogel for Prevention of Breast Cancer Recurrence with Theranostic and Mammoplastic Functions. Adv. Funct. Mater..

